# Checklist to the *Elatostema* (Urticaceae) of Vietnam including 19 new records, ten new combinations, two new names and four new synonyms

**DOI:** 10.7717/peerj.6188

**Published:** 2019-01-10

**Authors:** Long-Fei Fu, Alex Monro, Truong Van Do, Maxim S. Nuraliev, Leonid V. Averyanov, Fang Wen, Zi-Bing Xin, Tatiana V. Maisak, Andrey N. Kuznetsov, Svetlana P. Kuznetsova, Khang Sinh Nguyen, Yi-Gang Wei

**Affiliations:** 1 Guangxi Key Laboratory of Plant Conservation and Restoration Ecology in Karst Terrain, Guangxi Institute of Botany, Guangxi Zhuang Autonomous Region and Chinese Academy of Sciences, Guilin, China; 2 Identification and Naming Department, Herbarium, Royal Botanic Gardens, Kew, London, United Kingdom; 3 Department of Biology, Vietnam National Museum of Nature, Vietnam Academy of Science and Technology, Hanoi, Vietnam; 4 Joint Russian-Vietnamese Tropical Scientific and Technological Center, Hanoi, Vietnam; 5 Department of Higher Plants, Biological Faculty, M.V. Lomonosov Moscow State University, Moscow, Russia; 6 Komarov Botanical Institute of the Russian Academy of Sciences, St Petersburg, Russia; 7 Severtsov Institute of Ecology and Evolution Problems of the Russian Academy of Sciences, Moscow, Russia; 8 Institute of Ecology and Biological Resources, Vietnam Academy of Science and Technology, Hanoi, Vietnam

**Keywords:** Flora of Vietnam, Pellionia, Elatostemateae, Begonia, Orchidaceae, Taxonomy, Limestone, Karst, Indochina, Lectotypification

## Abstract

*Elatostema* (Urticaceae) comprises several hundred herbaceous species distributed in tropical and subtropical Africa, Asia, Australia and Oceania. The greatest species richness occurs on limestone karst in Southeast Asia. Taxonomic revisions of *Elatostema* are largely out of date and contradict each other with respect to the delimitation of *Elatostema* and *Pellionia.* Most herbaria in SE Asia and worldwide contain significant amounts of unidentified material. As part of a broader revision of *Elatostema* in SE Asia, we present an updated checklist for Vietnam based on field visits, a review of specimens in herbaria worldwide, a review of type material and nomenclature. We recognize 77 taxa (75 species and two infraspecific taxa) of *Elatostema* in Vietnam, 23 of which were previously ascribed to *Pellionia*. Nineteen of these are new records for the country, i.e., *E. attenuatoides*, *E. austrosinense*, *E. backeri*, *E. brunneinerve*, *E. crassiusculum*, *E. crenatum*, *E. fengshanense*, *E. glochidioides*, *E. malacotrichum*, *E. nanchuanense*, *E. oblongifolium*, *E. obtusum*, *E. oppositum*, *E. pergameneum*, *E. prunifolium*, *E. pseudolongipes*, *E. pycnodontum*, *E. salvinioides* and *E. xichouense*. We place *E. baviensis* in synonymy of *E. platyphyllum*, *E. colaniae* in synonymy of *E. myrtillus*, *P. macroceras* in synonymy of *E. hookerianum*, and *P. tetramera* in synonymy of *E. dissectum* for the first time. Fourteen taxa (18% of all the recognized taxa) are endemic to Vietnam, which makes *Elatostema* one of the richest genera for endemic species in this country; this level of endemism is comparable to levels observed in Orchidaceae. Our checklist suggests that the highest diversity and endemism of *Elatostema* occurs in northern Vietnam, and that there is the greatest floristic similarity of northern Vietnam to SW China. The relationship among floristic regions is also investigated. We could find no records of *Elatostema* for 33 out of 63 provincial units of Vietnam, including all the southernmost provinces. We propose that further studies on the diversity of *Elatostema* in central and southern Vietnam are severely needed.

## Introduction

*Elatostema* JR Forst. & G Forst. (Urticaceae) comprises several hundred species of succulent herbs and subshrubs that grow in shade in forests, gorges, stream sides and caves (Fu et al., 2017; Monro et al., 2018). There are currently 626 accepted names within the genus *Elatostema* (The Plant List, 2018). *Elatostema* is most diverse in subtropical and tropical climates and is characterized by apparently alternate (opposite but frequently with ephemeral and occasionally absent nanophylls), strongly asymmetrical leaves arranged distichously, small staminate flowers borne in dense cymes with receptacle-like involucres or paniculate cymes and minute pistillate flowers borne in dense capitate cymes, the bracts of which form a receptacle-like involucre (Tseng et al., 2019). As in most species in the tribe Elatostemateae (Conn & Hadiah, 2009), the stamens open explosively and the seeds are ejected by reflexive staminodes (Friis, 1989). *Elatostema* is distributed throughout tropical and subtropical Africa, Madagascar, Asia, Australia and Oceania, with highest diversity on limestone karst in China and Southeast Asia (Yahara, 1984; Lahav-Ginott & Cronk, 1993; Wang & Chen, 1995; Lin, Friis & Wilmot-Dear, 2003; Wang, 2014). The previous studies suggest that this diversity has been driven by the challenges of colonising karst substrates, which once overcome leads to species radiations (Chung et al., 2014; Fu et al., 2017), and that past temperature fluctuations and East Asian monsoons have driven the rates of plant diversification in karst ecosystems by accelerating the rate of karstification (Kong et al., 2017).

**Figure 1:**
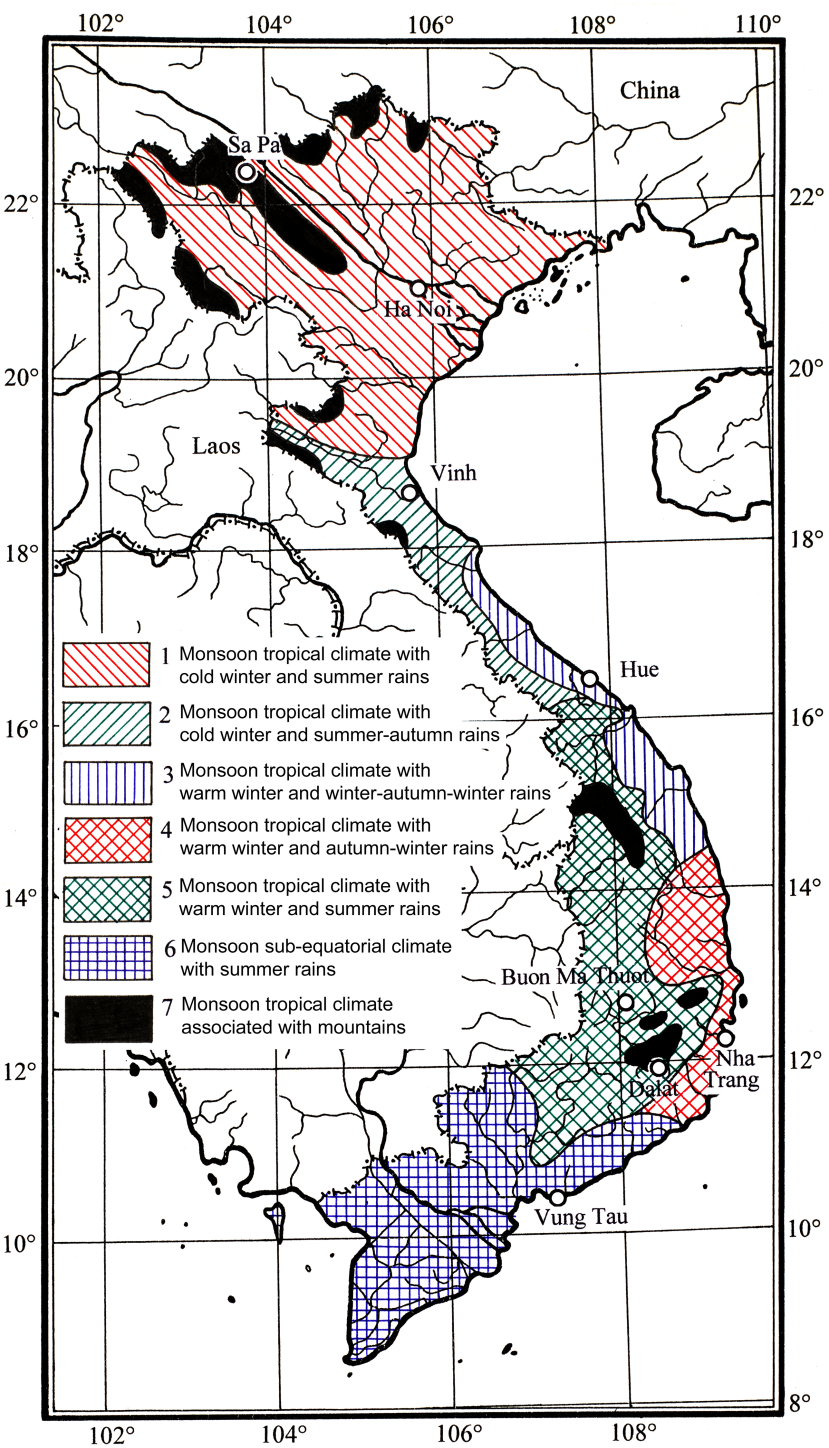
Climate map of Vietnam. The data based on Nguyen et al. (2000) showing seven types of climate in Vietnam.

**Figure 2:**
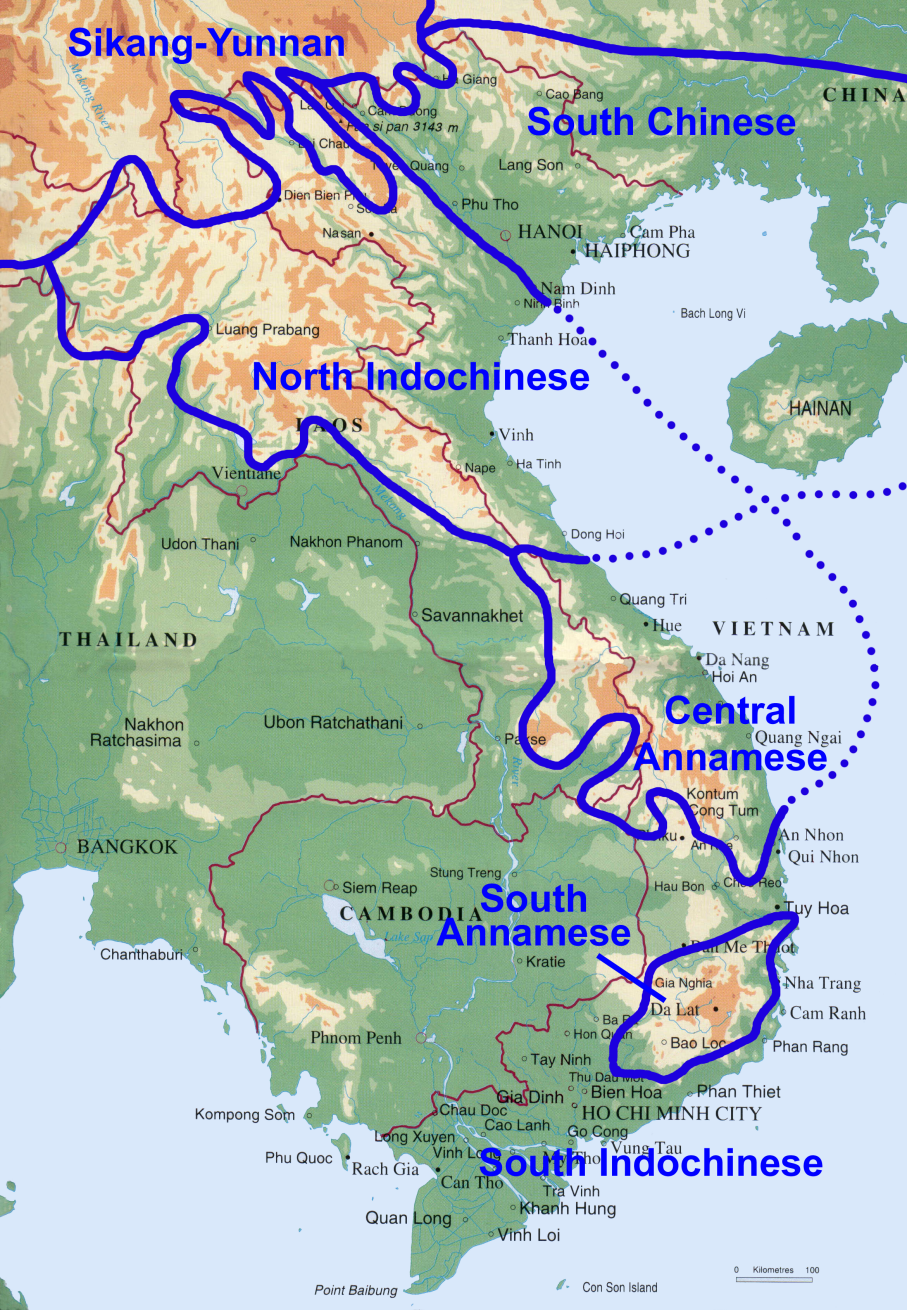
Map of floristic regions in Vietnam. The data modified from Averyanov et al. (2003c) showing six floristic regions in Vietnam.

Vietnam comprises 331,000 km^2^ of the eastern Indochinese Peninsula between latitudes 8° and 24°N, and longitudes 102° and 110°E. Mountains account for 40% of the surface area, and ca. 42% of the land is forested (De Queiroz et al., 2013). Vietnam’s climate divides broadly into humid subtropical in the north, monsoon in the centre and tropical savannah in the south (Peel, Finlayson & McMahon, 2007). However, on the basis of more detailed and accurate climate classification by Nguyen et al. (2000), seven types of climate are defined in Vietnam (Fig. 1) which are largely congruent with the floristic region classification (Takhtajan, 1978; Takhtajan, 1986; Averyanov et al., 2003a). Within Vietnam, six floristic regions have been delimited: the Sikang-Yunnan, South Chinese, North Indochinese, Central Annamese, South Annamese and South Indochinese Floristic Regions (Fig. 2). Vietnam also forms part of the Indo-Burma biodiversity hotspot and hosts 110 Key Biodiversity Areas (Mittermeier et al., 2004), and 65 Important Bird Areas (BirdLife International, 2002). Its flora comprises ca. 12,000 vascular plant species (Averyanov et al., 2003a), of which ca. 10% are endemic to the country (Pilgrim & Tu, 2007). Despite Vietnam’s rich plant diversity, it is still poorly sampled, with only ca. 14 specimen records per 100 km^2^ (Newman et al., 2007). A comparison of the 20 most species-rich families for neighbouring southern China places Urticaceae in the top ten most species-rich families (Zhu, 2017), yet it is absent from the top 20 families for Vietnam (Zhu, Yan & Qin, 2003). This suggests that Urticaceae are undersampled in Vietnam.

The delimitation of *Elatostema* has been controversial with respect to *Elatostematoides* C.B.Rob., *Pellionia* Gaudich. and *Procris* Comm. ex Juss. Using reconstruction of molecular phylogeny and re-evaluation of morphological characters of leaves, inflorescences and flowers, the latest study (Tseng et al., 2019) demonstrates that *Elatostema* is a monophyletic group which includes *Pellionia* but excludes *Procris*, *Elatostematoides* and *Pellionia repens* (Lour.) Merr.

Despite the large number of species descriptions and numerous revisions of *Elatostema* in China (Wang, 1980; Wang & Chen, 1995; Lin, Friis & Wilmot-Dear, 2003; Wei, Monro & Wang, 2011; Wang, 2014; Wang, 2016), there has been little work on the Southeast Asian taxa (Robinson, 1910; Gagnepain, 1930; Yahara, 1984; Beaman, 2001; Ho, 2003; Hiep, 2005). In addition, the most recent world-scale revision of this genus was undertaken in the 1930s before much of the region had been extensively covered by collections (Schroeter & Winkler, 1935; Schroeter & Winkler, 1936). Lack of taxonomic effort combined with high species diversity of *Elatostema*, high rate of species discovery and the frequency of point endemism has resulted in many Southeast Asian and world herbaria having significant numbers of undetermined *Elatostema* collections. The lack of a comprehensive species and distribution list for *Elatostema* in Vietnam therefore represents a barrier not only to its study but also to the assessment of extinction threats, conservation and sustainable use.

The first checklist of *Elatostema* in Vietnam (Gagnepain, 1930) documented 34 species (together with *Pellionia*). This was followed by two studies (Ho, 2003; Hiep, 2005) which documented 35 species and 32 species (together with *Pellionia*) respectively. Since that time further 27 species have been recorded from Vietnam in regional floras and checklists and other works (Wang, 1983; Wang & Chen, 1995; Lin, Friis & Wilmot-Dear, 2003; Lin, 2008; Lin et al., 2011; Fu et al., 2013; Fu et al., 2014a; Fu et al., 2014b; Lin, Duan & Bi, 2014a; Lin, Duan & Bi, 2014b; Wang, 2014; Do et al., 2017) and several species have had their circumscription changed (Shao, Lin & Duan, 2004; Wang, 2016). The generic placement of some species was also reevaluated (see above). Considering these numerous additions, our ongoing research in Southeast Asian *Elatostema*, the recently revised genus circumscription (Tseng et al., 2019) and the need to critically assess the extinction threat of the species we decided to produce an updated checklist to support further taxonomic and broader scientific studies.

## Materials & Methods

Fieldtrips were undertaken by two groups, a Sino-Vietnamese Group (SVG) and a Russian-Vietnamese Group (RVG). Between 2012 and 2017, SVG undertook five fieldtrips to the provinces or cities of Bac Kan, Cao Bang, Ha Giang, Ha Noi, Hai Phong, Hoa Binh, Nghe An, Lang Son, Lao Cai, Ninh Binh, Quang Binh, Thanh Hoa, Tuyen Quang and Vinh Phuc in northern Vietnam. During the course of these trips SVG made ca. 100 *Elatostema* collections. Over the last 30 years RVG has undertaken dozens of fieldtrips throughout Vietnam, which have resulted in ca. 200 *Elatostema* collections. In addition to field collecting we reviewed ca. 1,000 specimens of *Elatostema* at A, BM, GXMI, HN, IBK, IBSC, K, KUN, LE, MHA, MO, MPU, MW, P, PE, SING, TAI and VNMN. This was accompanied by a literature review which encompassed species protologues, the most recent monographs (Schroeter & Winkler, 1935; Schroeter & Winkler, 1936), and regional checklists (Robinson, 1910; Gagnepain, 1930; Backer, 1965; Yahara, 1984; Ho, 2003; Hiep, 2005; Wang, 2014; Wang, 2016). Using these sources of information, we have generated a preliminary species list. We then confirmed or refuted each name on the list against type material, either the original specimens or high resolution images of them, using the subscription-based full version of The JSTOR Global Plants portal (2017) and online databases of some well-digitized herbaria (e.g., E, K, P, PE) and consulting original description of each species so that only names for which a herbarium specimen or field image could be confidently associated with a type image or specimen or original description were included in the final checklist.

We used the morphological species concepts (Schroeter & Winkler, 1935; Schroeter & Winkler, 1936; Wang, 1980; Wei, Monro & Wang, 2011; Wang, 2014) to delimit species. These concepts place emphasis on peduncle bract shape and length, the number, morphology and arrangement of the bracts comprising the receptacle-like involucre, the number of bracteoles per flower and leaf lamina length/width ratios. Material was examined under a Changfang XTL-240 binocular microscope and Planapo lens at ×14 to ×90 magnifications.

For counting the diversity and endemism of *Elatostema* across floristic regions, we followed the concepts of Takhtajan (1978), Takhtajan (1986) and Averyanov et al. (2003a) to assign the location of each specimen to particular floristic region. The specimens without detailed localities that could not be assigned with confidence to a floristic region were not used in this analysis.

## Results

### Diversity & endemism

**Figure 3:**
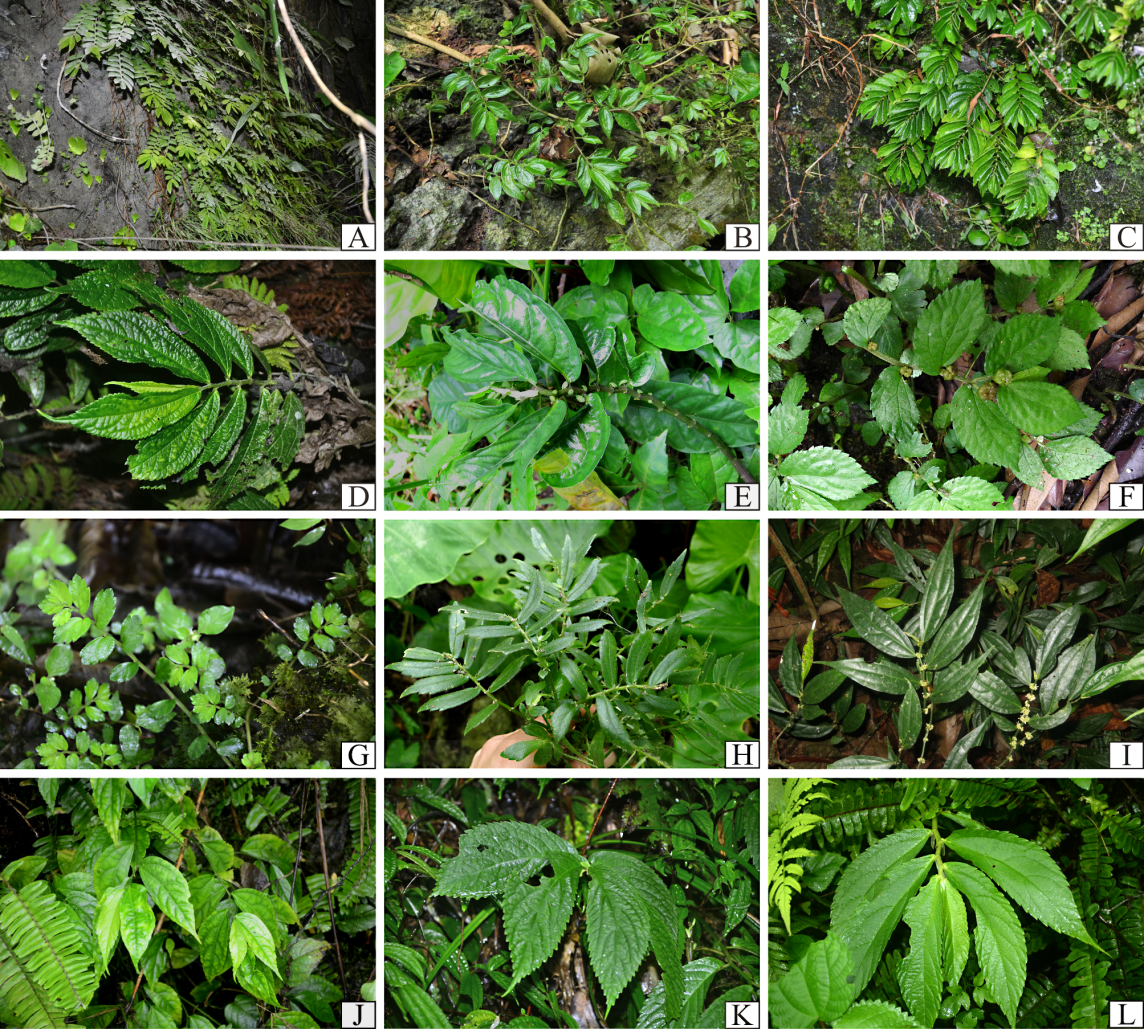
Plate I of representative *Elatostema* species in Vietnam: (A) *E. crassiusculum*; (B) *E. glochidioides*; (C) *E. hookerianum*; (D) *E. prunifolium*; (E), *E. arcuatobracteatum*; (F), *E. retrohirtum*; (G), *E. obtusum*; (H) *E. ramosum*; (I) *E. integrifolium*; (J) *E. fengshanense*; (K) *E. austrosinense*; (L) *E. malacotrichum*.

**Figure 4:**
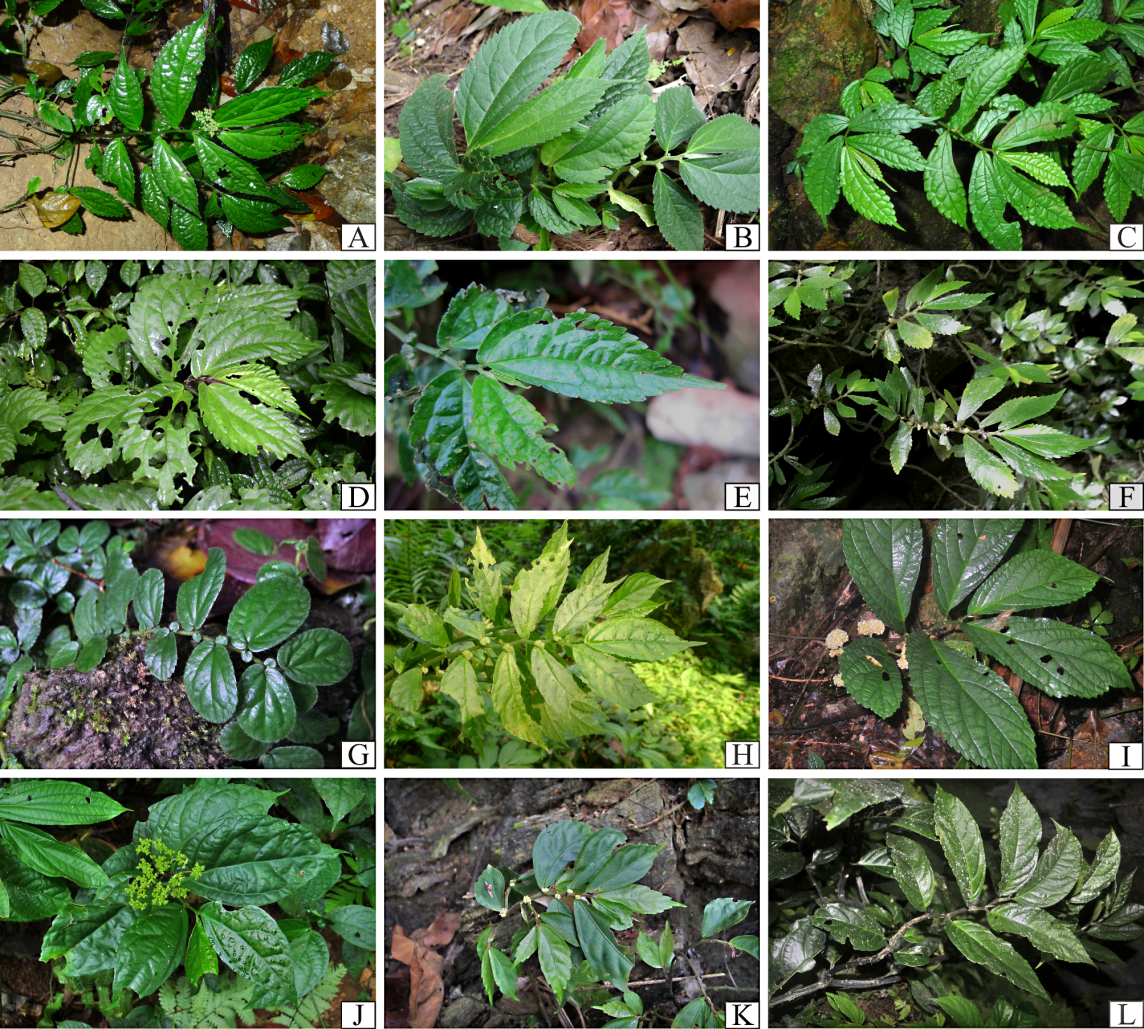
Plate II of representative *Elatostema* species in Vietnam: (A) *E. heterolobum*; (B), *E. atroviride*; (C) *E. tenuicaudatum*; (D) *E. nasutum*; (E) *E. cyrtandrifolium*; (F) *E. backeri*; (G), *E. veronicoides*; (H) *E. dissectum*; (I) *E. atropurpureum*; (J) *E. paucidentatum*; (K) *E. lineolatum*; (L) *E. xanthophyllum*.

**Figure 5:**
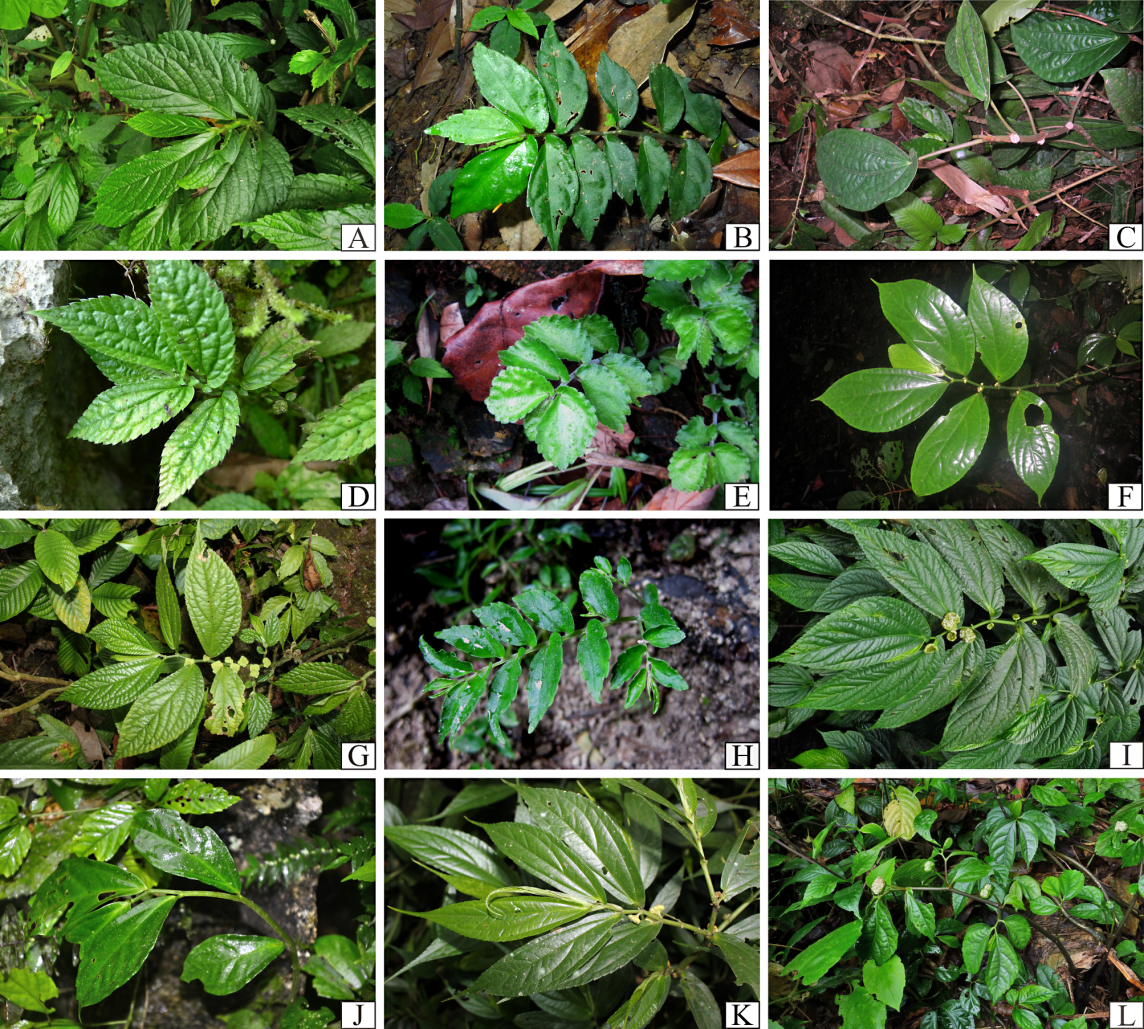
Plate III of representative *Elatostema* species in Vietnam: (A), *E. caulialatum*; (B), *E. radicans*; (C), *E. tsoongii*; (D), *E. sinense*; (E), *E. brevifolium*; (F), *E. macintyrei*; (G), *E. balansae*; (H), *E. myrtillus*; (I) *E. platyphyllum*; (J) *E. petelotii*; (K) *E. longistipulum*; (L) *E. pseudolongipes*.

We document 77 taxa of *Elatostema* in Vietnam, 14 taxa (18%) of which are endemic to the country. These taxa comprise 75 species, two of which are represented each by two taxa of a lower level (varieties and subspecies). Table 1 provides a summary of the species richness, national endemism, and new national records identified here, per floristic region. Figures 3–5 provide field images of a representative sample of these species.

**Table 1:** The species richness, endemism, and new records identified per Floristic Region.

Floristic region	Species no.	Endemic species no.	New records no.
Sikang-Yunnan Floristic Region	36	2	9
South Chinese Floristic Region	54	5	12
North Indochinese Floristic Region	36	1	4
Central Annamese Floristic Region	16	2	0
South Annamese Floristic Region	7	0	0
South Indochinese Floristic Region	0	0	0

**Figure 6:**
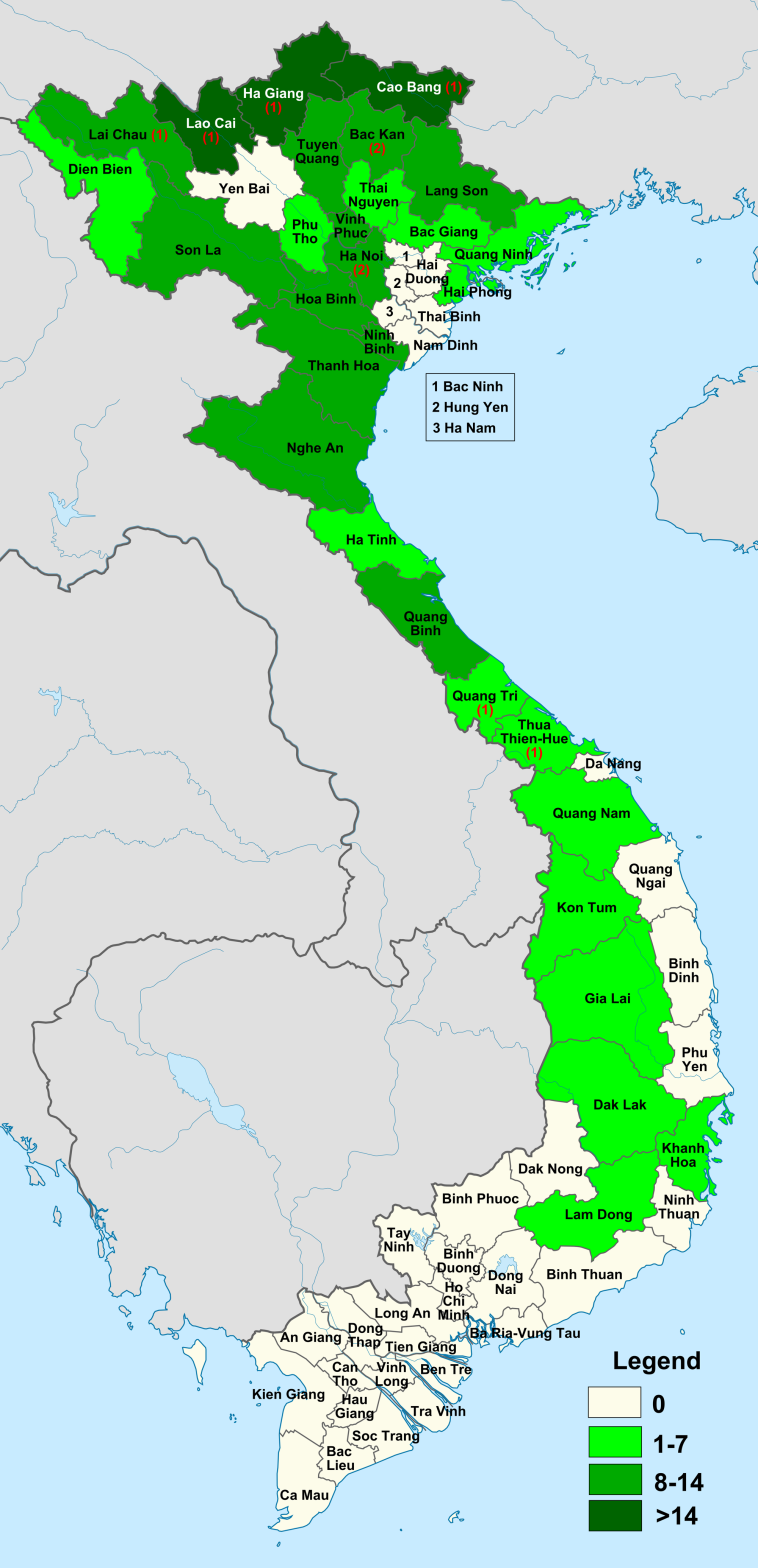
Variation in *Elatostema* species richness and endemism across provinces of Vietnam. The tones of green indicate number of species per province. The red figures in brackets indicate number of national endemic species recorded for a province where it is non-zero.

We document the greatest species richness in northern Vietnam (Cao Bang, Ha Giang, Lao Cai provinces) and the lowest species richness in eight scattered provinces across northern to central Vietnam and 25 southernmost provincial units where no *Elatostema* species have been documented: Bac Ninh, Ha Nam, Hai Duong, Hung Yen, Nam Dinh, Thai Binh, Yen Bai (North), Da Nang City (Central) and An Giang, Ba Ria-Vung Tau, Bac Lieu, Ben Tre, Binh Dinh, Binh Duong, Binh Phuoc, Binh Thuan, Ca Mau, Can Tho City, Dak Nong, Dong Nai, Dong Thap, Hau Giang, Ho Chi Minh City, Kien Giang, Long An, Ninh Thuan, Phu Yen, Quang Ngai, Soc Trang, Tay Ninh, Tien Giang, Tra Vinh, and Vinh Long (South, Fig. 6). We recovered the greatest number of species endemic to the country in northern Vietnam (particularly Bac Kan province and Ha Noi City) (Fig. 6).

### Relationship among floristic regions

**Figure 7:**
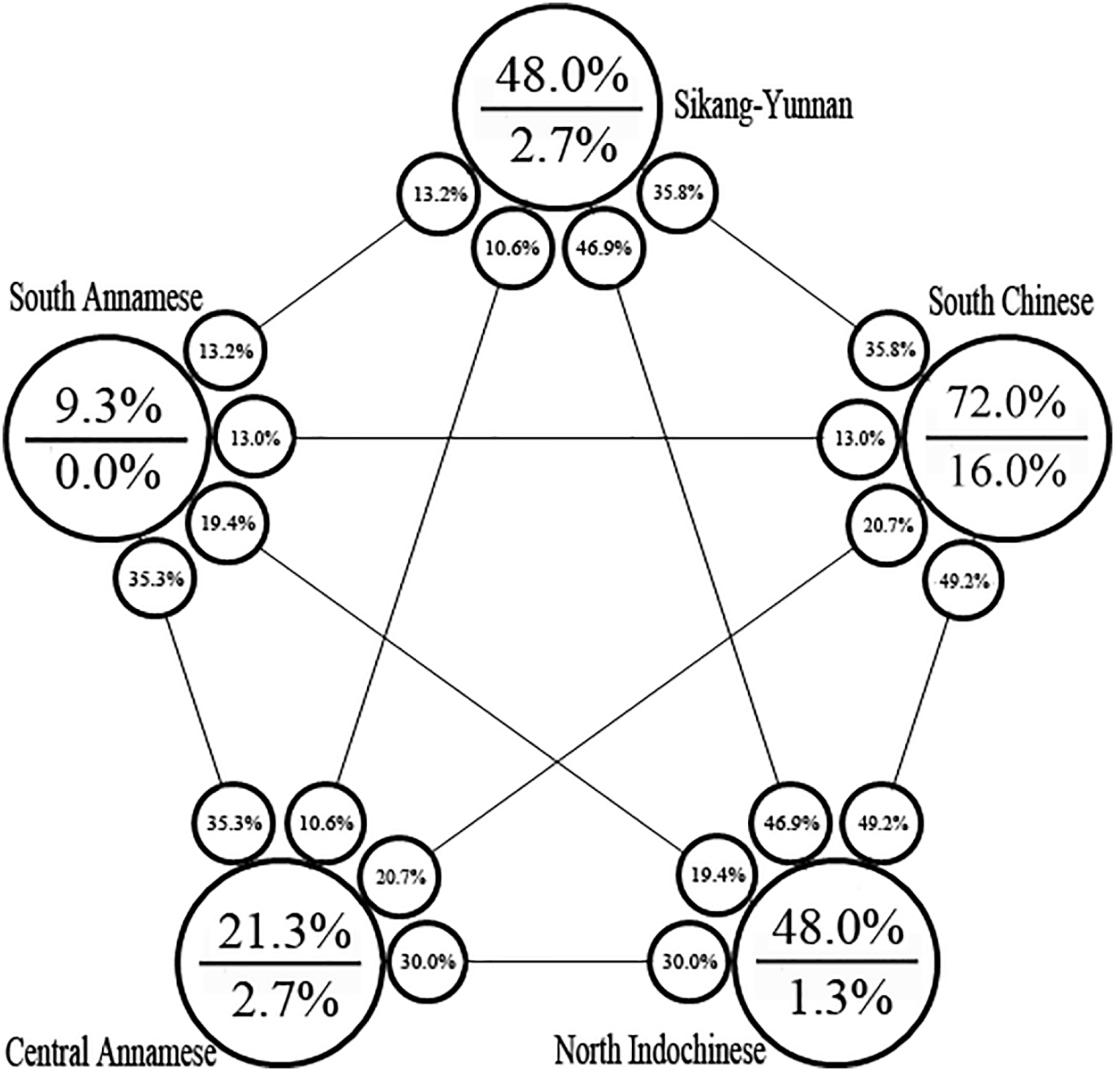
Proportion of diversity and endemism and the relationship of the floristic regions. Large circles designate floristic regions. Percentage numbers above the line in the large circles indicate the relative proportion of diversity occurring in a certain region (within Vietnam); percentage numbers below the line indicate the relative proportion of national endemics occurring in a certain region; percentage numbers in the small circles indicate the relative proportion of co-exsisted species occurring in the pairs of region connected by the line and show their relative floristic relationships.

Figure 7 provides the proportion of diversity and endemism and the relationship of each floristic region. The order of the proportion of diversity of two floristic regions is the following: South Chinese–North Indochinese (49.2%) >Sikang-Yunnan–North Indochinese (46.9%) >Sikang-Yunnan–South Chinese (35.8%) >Central Annamese–South Annamese (35.3%) >North Indochinese–Central Annamese (30.0%) >South Chinese–Central Annamese (20.7%) >North Indochinese–South Annamese (19.4%) >Sikang-Yunnan–South Annamese (13.2%) >South Chinese–South Annamese (13.0%) >Sikang-Yunnan–Central Annamese (10.6%).

### New records, new combinations, new names & synonymy

We found 19 new records of *Elatostema* for Vietnam, i.e., *E. attenuatoides* W.T.Wang, *E. austrosinense* Y.H.Tseng & A.K.Monro *nom. nov.*, *E. backeri* H.Schroet., *E. brunneinerve* W.T.Wang, *E. crassiusculum* W.T.Wang, *E. crenatum* W.T.Wang, *E. fengshanense* W.T.Wang & Y.G.Wei, *E. glochidioides* W.T.Wang, *E. malacotrichum* W.T.Wang & Y.G.Wei, *E. nanchuanense* W.T.Wang, *E. oblongifolium* Fu, *E. obtusum* Wedd., *E. oppositum* Q.Lin & Y.M.Shui, *E. pergameneum* W.T.Wang, *E. prunifolium* W.T.Wang, *E. pseudolongipes* W.T.Wang & Y.G.Wei, *E. pycnodontum* W.T.Wang, *E. salvinioides* W.T.Wang, and *E. xichouense* W.T.Wang. All new records came from the northern provinces: Cao Bang, Ha Giang, Lao Cai, Dien Bien, Lang Son, Phu Tho, Bac Kan, Hoa Binh and Ninh Binh (Table 1); all of them are also known from the neighbouring regions of China, i.e., Guangxi, Guizhou and Yunnan. Of the Vietnamese provinces, Cao Bang and Ha Giang had the greatest number of new records.

We place *Elatostema baviensis* Gagnep. in synonymy of *E. platyphyllum* Wedd. based on the characters of large lamina (10–25 × 4–7.5 cm) with auriculate base, large stipule (15–25 × 3–5.5 mm) and pistillate inflorescence with short peduncle and obvious receptacle. Then, we place *E. colaniae* Gagnep. in synonymy of *E. myrtillus* Hand.-Mazz. based on the characters of small lamina (13–28 × 6–10 mm) with auriculate base and furfuraceous stem base. We also place *Pellionia macroceras* Gagnep. in synonymy of *E. hookerianum* Wedd. based on the characters of falcate lamina with cordate base. Finally, we place *P. tetramera* Gagnep. in synonymy of *E. dissectum* Wedd. based on the characters of oblong-lanceolate lamina and staminate inflorescence with long peduncle. In addition and in line with the study by Tseng et al. (2019), we transfer all previously known *Pellionia* species found in Vietnam (except for *P. repens*) to *Elatostema* including ten new combinations and two new names proposed accordingly.

## Discussion

### Diversity & endemism

Our checklist of *Elatostema* in Vietnam doubles the number of species cited in the most recent checklist (Hiep, 2005) bringing the total diversity to 77 taxa (75 species). This is the product of both recent checklists for the region, field collecting and herbarium specimen examination, aided by the availability of protologues, type images and other collections online (e.g., The JSTOR Global Plants portal, 2017; Seregin, 2018).

We also document that species richness and endemism increase with latitude, away from the equator. Whilst counter-intuitive, a similar pattern has been documented for the diversity of *Begonia* L. (Averyanov & Nguyen, 2012) and Orchidaceae (Averyanov et al., 2003a). This is likely a reflection of the distribution of karst in Vietnam with which *Elatostema* (Wang, 2014), *Primulina* Hance (Kong et al., 2017) and *Begonia* (Chung et al., 2014) species richness in Southeast Asia is strongly associated rather than *Elatostema*’s preference for a more seasonal or cooler climate. Given the total absence of *Elatostema* records from Vietnam’s southernmost provinces this pattern may also have been over-emphasised by unequal sampling effort across the country as well as by unequal distribution of intact vegetation. Indeed, the southernmost provinces are largely plain and therefore occupied by agricultural landscapes.

The documented 18% level of endemism of the *Elatostema* taxa in Vietnam is nearly twice the average for the country’s flora (10%, Pilgrim & Tu, 2007) and comparable to that in the most species-rich family Orchidaceae (19%, Averyanov et al., 2003a). However, this level is still lower than the corresponding level in Guizhou (39%), Guangxi (64%) and Yunnan (71%) of China, which are well documented (Wang, 2014). As in China, an association with karst is likely the driver for much of this diversity indicating Vietnam’s capacity of higher endemism which is likely to be revealed when *Elatostema* is comprehensively documented. Current researches (Chung et al., 2014; Kong et al., 2017) suggest that rates of karstification over time, and likely in the Miocene, associated with seasonal climate and temperature, may have driven speciation on karst. Species that were able to adapt to what is a relatively inhospitable substrate speciating as karst habitat was formed. It is notable that of the documented genera that have ‘radiations’ associated with karst, i.e., *Begonia*, *Impatiens* L., *Primulina* and *Elatostema*, all are mostly herbaceous, three of the four are succulent, three of the four are insect-pollinated and all have seeds that are abiotically rather than animal dispersed.

### Comparison of diversity among floristic regions, and reasons of its differences

Among the six floristic regions delimited in Vietnam (see Fig. 2; Takhtajan, 1978; Takhtajan, 1986; Averyanov et al., 2003a), we found the greatest species richness and endemism in the South Chinese Floristic Region (Figs. 6 and 7; see Table 1). We attribute this to widespread presence of karst, monsoon tropical climate with cold winter and summer rains and tropical forests (Fig. 2) with which *Elatostema* species richness and endemism is known to be associated (Wang, 2014). For example, the checklist of *Elatostema* in China documented 184 out of the 280 species recorded as restricted to karst, many of which are range-restricted endemics (Wang, 2014). A similar pattern in species richness in Vietnam has been documented for *Begonia* by Averyanov & Nguyen (2012) who also ascribe part of this richness to the presence of karst in this floristic region.

The second highest species diversity and endemism occurred in the Sikang-Yunnan Floristic Region. Notably, this region occupies one of the smallest areas in Vietnam, comparable only with the area of the South Annamese Region; this indicates extraordinarily high average *Elatostema* species density in the Sikang-Yunnan Floristic Region. This region lies within the SSE extension of the Himalayan Highlands. It can be compared with another representative Himalayan area, i.e., Xizang province, which is the fourth most species-rich province for *Elatostema* in China (Wang, 2014). Within the Sikang-Yunnan Floristic Region, 17 species of *Elatostema* (22.7% of the species that we document for Vietnam) occur in Hoang Lien Son Range of Lao Cai province. It is notable that the Sikang-Yunnan Floristic Region is the only one within the territory of Vietnam which represents the Holarctic floristic kingdom, while the other five regions belong to the Paleotropical floristic kingdom (Takhtajan, 1978; Takhtajan, 1986; Averyanov et al., 2003a; Averyanov et al., 2003b). This explains the high diversity of *Elatostema* in this region, as this genus is also known to be mostly associated with the southern part of the Holarctic kingdom (Wang, 2014).

The next most important floristic region for species richness and endemism of *Elatostema* in Vietnam is the North Indochinese Floristic Region (Fig. 7; Table 1). It possesses the same species number as the previous region and a single national endemic species instead of two. This region covers a chain of continuous limestone plateaux (Averyanov et al., 2003a). Monsoon tropical climate with cold winter and summer rains is typical of the largest part of the northern portion (Fig. 2) which is similar to those found in the South Chinese Region. Therefore, the diversity and endemism of this floristic region share similar pattern to the South Chinese Region which is congruent with our result that nearly half of species (49.2%) co-existed. This region also includes the southern extension of the Himalayan Highlands and our result revealed 46.9% co-existed species (Fig. 7) between it and Sikang-Yunnan Region, which also suggests their similarity. It should also be taken into account that the North Indochinese Floristic Region occupies almost the largest area within Vietnam, comparable only with the area of South Indochinese Region. Therefore the high species diversity in this region is also an effect of its large territory.

We found low species richness and endemism in the Central Annamese Floristic Region (Fig. 6; see Table 1). The representative provinces of this floristic region are Quang Tri and Thua Thien-Hue which contain karst mountains and a climate very similar to that of the North Indochinese Floristic Region (Averyanov et al., 2003a); besides, 30.0% of species co-occurred in these two floristic regions (Fig. 7). We would therefore expect species richness and endemism in the Central Annamese Floristic Region to be similar to those in the North Indochinese Floristic Region rather than lower (Table 1). We would also expect the diversity to be higher (rather than the same) in the Central Annamese Floristic Region than in the South Annamese Floristic Region, as the latter lacks karst and experiences drier climate. We also found 31.3% species shared between the Central Annamese and South Annamese Floristic Regions. Therefore, we propose that both of these phenomena reflect a lack of collecting effort.

The South Indochinese Floristic Region, the largest in Vietnam (Fig. 2), comprises lowland area of 23 provincial units and yet we document no species records from all of them (Fig. 6). This is unlikely to be due to the absence of karst, edaphic factors or climate alone. Whilst this floristic region has little karst (Kien Giang province only), it does have multiple climate types (Fig. 1) and significant forest cover (Global Forest Watch, 2014) which elsewhere in SE Asia would support a diversity of *Elatostema* species (Robinson, 1910; Conn, 2008). Again, we propose that this absence of records is the result of a lack of collecting effort rather than a consequence of total absence of this genus. This inference is also supported by the absence of known *Elatostema* collections from several Vietnamese provinces surrounded by areas with rather high diversity of this genus: Yen Bai, Thai Binh, Nam Dinh, Bac Ninh, Hai Duong, Hung Yen, Ha Nam, Da Nang, Quang Ngai and Binh Dinh. We therefore propose that South Indochinese Floristic Region should be of a high priority for subsequent studies of *Elatostema* in Vietnam.

## Conclusions

This study combines taxonomic and field expertise from China, Russia, the United Kingdom and Vietnam. It has strongly benefited from the availability of type images online which has accelerated the process of identification and the evaluation of taxon names. Ongoing research to document the floras of Cambodia, Laos, Thailand and Vietnam provides a huge opportunity for the taxonomy of *Elatostema*. Once combined with the completed Flora of China and Flora Malesiana accounts our updated checklist for Vietnam will fill a significant knowledge gap for this species-rich genus and lay the foundations for a global checklist. This fourth checklist for Vietnam not only doubles estimates of the diversity of the genus, it also identifies major knowledge gaps for the country, i.e., central Vietnam and, most notably, southern Vietnam. We propose that greater sampling effort of the flora of central Vietnam and southern Vietnam will result in a number of new additions to the flora of the country.

## Appendix. Checklist of *Elatostema* in Vietnam

Species are listed in alphabetical order. Basionyms and synonyms are cited on a separate line in alphabetical order. This is followed by the species’ global distribution, elevation range within Vietnam and the exsiccatee seen for this study. New records for Vietnam are denoted by an ‘*’ next to the name.

**Elatostema acuminatum** (Poir.) Brongn.

*Procris acuminata* Poir*.*

*Boehmeria acuminata* Pers., *Elatostema lowii* Stapf., *Elatostema membranaceum* Hassk., *Elatostema membranifolium* Kurz, *Langeveldia acuminata* Gaudich., *Ramium acuminatum* (Pers.) Kuntze.

Bhutan, China, Indonesia, Malaysia, Myanmar, Nepal, Sikkim, Thailand, Vietnam; 400–1,650 m.

**Cao Bang Province**: Ha Lang District, Dong Loan Municipality, 22°46′N103°44′E, *L. Averyanov et al. CBL603* (LE); **Ha Giang Province**: Bac Me District, Phieng Luong Municipality, 22°39′29″N 105°19′35″E, *L. Averyanov et al. HAL6540* (LE); Quan Ba District, Can Ty Municipality, 23°04′N 104°59′E, *N.T. Hiep et al. NTH3622* (LE); Vi Xuyen District, Cao Bo Municipality, Tam Ve Village, 22°46′18″N 104°49′01″E, *D.K. Harder et al. 5615* (K, MO); **Ha Noi City**: Ba Vi District, Ba Vi National Park, 21°04′22″N 105°21′53″E, *L.F. Fu et al. VMN_CN35* (IBK, VNMN); **Quang Binh Province**: Minh Hoa District, Dan Hoa Municipality, 17°45′28″N 105°46′14″E, *L. Averyanov et al. HAL12205* (LE), 17°46′34″N 105°48′56″E, *L. Averyanov et al. HAL12548* (LE); **Vinh Phuc Province**: Tam Dao District, *L. Averyanov et al. LX-VN864* (LE).

**Elatostema acutidentatum** (W.T.Wang) Y.H.Tseng & A.K.Monro, *comb. nov.*

Basionym: *Pellionia acutidentata* W.T.Wang, Bull. Bot. Lab. N.-E. Forest. Inst., Harbin 6: 53. 1980. Type: China. Yunnan, Xichou county, Fadou village, *C.W. Wang & Y. Liu 85536* (holotype: PE!).

China, Vietnam; 1,400–1,900 m.

**Cao Bang Province**: Nguyen Binh District, Nguyen Binh Municipality, 22°37′N 105°52′E, *L. Averyanov et al. CBL195* (LE, MO); **Lai Chau Province**: Than Uyen District, Ho Mit Municipality, 22°06′N 103°52′E, *N.T. Hiep et al. NTH2842* (LE); **Lao Cai Province**: Sa Pa District, *Anon. TV-284* (HN); Van Ban District, 21°59′00″N 104°02′00″E, *L. Averyanov et al. HAL2215* (LE).

**Elatostema albopilosum** W.T.Wang

a. **Elatostema albopilosum** var. **vicinum** L.D.Duan & Y.Lin

Endemic; 200–400 m.

**Bac Kan Province**: Cho Don District, Xuan Lac Commune, 22°21′91″N 105°32′95″E, *L.Q. Li et al. 0432* (PE); **Tuyen Quang Province**: Na Hang District, *H.N. Qin et al.* 319 (PE).

This variety is very similar to *E. albopilosum* W.T.Wang var. *albopilosum* and further study may result in it being treated as a synonym.

**Elatostema arcuatobracteatum** L.F.Fu, V.T.Do & C.X.He

Endemic; ca. 500 m.

**Ha Giang Province**: Bac Me District, Bac Me Nature Reserve, 22°45′15″N 105° 13′43″E, *L.F. Fu et al. VMN_CN219* (IBK, VNMN).

**Elatostema atropurpureum** Gagnep.

China, Vietnam; 300–1,150 m.

**Cao Bang Province**: Nguyen Binh District, Phia Oac—Phia Den National Park, *F. Wen et al. VMN_CN532* (IBK, VNMN); **Ha Noi City**: Ba Vi District, Ba Vi Mountain, *B. Balansa 2534* (P), 21°04.569′N 105°21.765′E, *N.A. Vislobokov & S.P. Kuznetsova s.n.* (MW); **Ha Giang province**: Quan Ba District, Tung Vai Municipality, Thang village, 23°03′13″N 104°51′49″E, *L. Averyanov VR558* (LE); **Lao Cai Province**: Sa Pa District, *Sino-Vietnam Expedition 336* (IBSC, PE); Van Ban District, Hoang Lien—Van Ban Nature Reserve, *L.F. Fu et al. VMN_CN920* (IBK, VNMN); *P.A. Petelot 5100* (P); **Ninh Binh Province**: Nho Quan District, Cuc Phuong National Park, *F. Wen et al. VMN_CN600*, *VMN_CN606* (IBK, VNMN); **Phu Tho Province**: Thanh Son District, Xuan Son Municipality, Xuan Son National Park, 21°08′00″N 104°55′55″E, *M.S. Nuraliev & N.A. Vislobokov 1105* (IBK, MW).

**Elatostema atroviride** W.T.Wang

*Elatostema atroviride* var*. lobulatum* W.T.Wang, *E. leiocephalum* W.T.Wang, *E. papilionaceum* W.T.Wang*.*

China, Vietnam; 200–1,500 m.

**Cao Bang Province**: Ha Quang District, Pac Bo, *L.F. Fu et al. VMN_CN783* (IBK, VNMN); **Ha Giang Province**: Yen Minh District, Lao Va Chai Municipality, 23°07′N 105°08′E, *N.T. Hiep et al. NTH3448* (LE); **Hai Phong City**: Cat Hai District, *Y.G. Wei et al. VMN_CN14* (IBK, VNMN); Cat Hai District, Cat Ba Island, *N. Arnautov et al. LX-VN3626, LX-VN3774, LX-VN3793, LX-VN3834* (LE); between Meo Vac District and Dong Van District, 23°15′16″N 105°23′12″E, *L.F. Fu et al. VMN_CN190* (IBK, VNMN), 22°45′23″N 105°13′46″E, *L.F. Fu et al. VMN_CN218* (IBK, VNMN); **Hoa Binh Province**: Mai Chau District, Pa Co Municipality, 20°44′32.3″N 104°56′26.9″E, *V.X. Phuong et al. HNK639* (K); **Nghe An Province**: Quy Chau District, Pu Huong Natural Reserve, *F. Wen et al. VMN_CN478* (IBK, VNMN); Con Cuong District, Cay Da Valley, Truong Huong Hamlet, Yen Khe Commune, 19°00′39.3″N 104°51′38″E, *V.D. Nguyen et al. HNK2960* (K); **Ninh Binh Province**: Nho Quan District, Cuc Phuong National Park, *J.M. Hu Hu2177* (TAI); **Quang Binh Province**: Minh Hoa District, Thong Hoa Municipality, 17°40′N 105°57′E, *L. Averyanov et al. VH4797* (LE); Tuyen Hoa District, Lam Hoa Municipality, 17°55′41″N 105°49′43″E, *L. Averyanov et al. CPC2620* (LE); **Son La Province**: Moc Chau District, Van Ho Municipality, Hua Tat Village, 20°46′18″N 104°47′45″E, *D.K. Harder et al. 5774* (HN, K, MO), 20°46′18″N 104°47′29″E, *N.T. Hiep et al. HAL9311* (LE); **Thanh Hoa Province**: Quy Chau District, Pu Huong Natural Reserve, *F. Wen et al. VMN_CN427* (IBK, VNMN); Thuong Xuan District, Bat Mot Municipality, 20°01′12″N 104°57′48″E, *L. Averyanov et al. CPC6655* (LE).

***Elatostema attenuatoides** W.T.Wang

China, Vietnam; 500–800 m.

**Cao Bang Province**: Ha Lang District, An Lac Municipality, 22°43′N 103°36′E, *L. Averyanov et al. CBL786* (LE, MO); Tra Linh District, Quoc Toan Municipality, 22°45′15″N 106°17′54″E, *D.K. Harder et al.* 4336 (K); 22°45′30″N 106°17′22″E, *L. Averyanov et al. VH4854* (HN, LE, MO), *VH4949* (LE, MO); 22°45′N 106°19′E, *L. Averyanov et al. CBL1256* (LE, MO).

***Elatostema austrosinense** Y.H.Tseng & A.K.Monro *nom. nov.*

non *E. macrophyllum* Brongn.

*Pellionia macrophylla* W.T.Wang, Acta Bot. Yunnan. 5: 271. 1980. Type: China. Yunnan, Luchun county, Huanglianshan, *D.D. Tao 845* (holotype: KUN!).

China, Vietnam; ca. 1,650 m.

**Lao Cai Province**: Sa Pa District, Hoang Lien National Park, *L.F. Fu et al. VMN_CN884* (IBK, VNMN); Van Ban District, 21°58′42″N 104°00′37″E, *L. Averyanov et al. HAL2072, HAL2088* (LE).

We selected ‘*austrosinense*’ as epiphet as it coincides with the origin of its distribution area, South China.

**Elatostema backanense** (Gagnep.) Y.H.Tseng & A.K.Monro, *comb. nov.*

Basionym: *Pellionia backanensis* Gagnep., Bull. Soc. Bot. France 75: 918. 1928. Type: Vietnam. Bac Kan, Yen Lac District, *P.A. Eberhardt 4669* (holotype: P!).

Endemic; elevation unknown.

**Bac Kan Province**: Yen Lac District, *P.A. Eberhardt 4669* (P).

***Elatostema backeri** H.Schroet.

China, Indonesia, Vietnam; elevation unknown.

**Cao Bang Province**: Bao Lac District, Dinh Phung Municipality, *L.F. Fu et al. VMN_CN814* (IBK, VNMN); Bao Lam District, Tan Viet, Na Dang, *L.F. Fu et al. VMN_CN829* (IBK, VNMN); **Thanh Hoa Province**: Quan Hua District, Pu Luong Nature Reserve, *F. Wen et al. VMN_CN551* (IBK, VNMN).

**Elatostema balansae** Gagnep.

*Elatostema balansae* var. *hispidum* W.T.Wang, *E. platyphyllum* Wedd. var. *balansae* (Gagnep.) Yahara*.*

China, Malaysia, Thailand, Vietnam; 1,100–1,700 m.

**Cao Bang Province**: Nguyen Binh District, Phia Oac—Phia Den Nature Reserve, 22°37′49″N 105°53′11″E, *L.F. Fu et al. VMN_CN170* (IBK, VNMN); **Ha Giang Province**: between Meo Vac District and Dong Van District, 23°09′16″N 105°24′52″E, *L.F. Fu et al. VMN_CN192* (IBK, VNMN); **Hoa Binh Province**: Mai Chau District, Hang Kia Municipality, 20°43′39″N 104°53′43″E, *N.T. Hiep et al. HAL751* (HN); **Lai Chau Province**: Sin Ho District, Ta Ngau Municipality, 22°18′N 103°15′E, *N.T. Hiep et al. NTH2783* (LE); **Lam Dong Province**: Lac Duong District, Da Chais Municipality, 12°08′N 108°39′E, *L. Averyanov et al. VH4511* (LE, MO); Lac Duong District, Da Chais Municipality, Bi Doup mountains, 12°11′07.0″N 108°42′55.9″E, *A.N. Demidova & N.G. Prilepsky 431* (MW); **Son La Province**, Yen Chau District, Muong Lum Municipality, On Oc Village, 20°59′44″N 104°30′03″E, *D.K. Harder et al. 7229* (HN).

**Elatostema brachyodontum** (Hand.-Mazz.) W.T.Wang

*Elatostema ficoides* Wedd. var. *brachyodontum* Hand.-Mazz.

China, Vietnam; elevation unknown.

**Province unknown**: Phuong-Lam, *B. Balansa 2535* (P).

**Elatostema brevifolium** (Benth.) Hallier f.

*Pellionia brevifolia* Benth.

*Amaranthus polychroa* Raeusch., *Elatostema radicans* var. *minimum* (Makino) H.Schroet. *Pellionia minima* Makino*.*

China, Japan, Vietnam; 60–1,900 m.

**Cao Bang Province**: Nguyen Binh District, Phia Oac—Phia Den Nature Reserve, 22°36′29″N 105°52′15″E, *L.F. Fu et al. VMN_CN161* (IBK, VNMN); **Lao Cai Province**: Sa Pa District, Hoang Lien National Park, Ton Forest Station, *L.F. Fu et al. VMN_CN915* (IBK, VNMN); Sa Pa District, San Sa Ho Municipality, 22°21′10″N 103°46′30″E, *M.S. Nuraliev & D.D. Sokoloff 263* (IBK, K, MW).

***Elatostema brunneinerve** W.T.Wang

China, Vietnam; 1,200–1,400 m.

**Ha Giang province**: Quan Ba District, Tung Vai Municipality, Thang village, 23°03′42″N 104°50′42″E, *L. Averyanov et al. VR591* (LE).

**Elatostema caulialatum** (S.Y.Liou) Y.H.Tseng & A.K.Monro, *comb. nov.*

Basionym: *Pellionia caulialata* S.Y.Liou, Guihaia 3(4): 317. 1983. Type: China. Guangxi, Guiping County, Dapingshan, *S.Y. Liu et al. D00322* (holotype: GXMI!, isotypes: PE!).

China, Vietnam; 200–415 m.

**Bac Kan Province**: Cho Don District, Xuan Lac Community, 22°21.91′N 105°32.95′E, *L.Q. Li et al. 0386* (HN); Na Ri District, Kim Hy Nature Reserve, 22°17′54″N 106°01′40″E, *L.F. Fu et al. VMN_CN128* (IBK, VNMN); **Ha Giang Province**: Meo Vac District, *L.F. Fu et al. VMN_CN859* (IBK, VNMN); **Thai Nguyen Province**: Vo Nhai District, Truong Lung Municipality, 21°48.75′N 105°58.09′E, *L.Q. Li et al. 0142* (HN); **Tuyen Quang Province**: Na Hang District, *Anon. TV-93* (HN).

**Elatostema chapaense** (Gagnep.) Y.H.Tseng & A.K.Monro, *comb. nov.*

Basionym: *Pellionia chapaensis* Gagnep., Bull. Soc. Bot. France 75: 920. 1928. Type: Vietnam. Lao Cai, Sa Pa District, *H. Lecomte & A. Finet 415* (holotype: P!).

Endemic; elevation unknown.

**Lao Cai Province**: Sa Pa District, *H. Lecomte & A. Finet 415* (P).

**Elatostema cochinchinense** (Gagnep.) Y.H.Tseng & A.K.Monro, *comb. nov.*

Basionym: *Pellionia cochinchinensis* Gagnep., Bull. Soc. Bot. France 75: 921. 1928. Type: Vietnam. Haut Donnai, Con-heu Mountain, *L. Pierre 4814* (holotype: A!).

Endemic; elevation unknown.

**Province unknown**: Haut Donnai, Con-heu Mountain, *L. Pierre 4814* (A).

***Elatostema crassiusculum** W.T.Wang

China, Vietnam; ca. 200 m.

**Cao Bang Province**: Bao Lam District, Nam Quang, *L.F. Fu et al. VMN_CN838* (IBK, VNMN).

***Elatostema crenatum** W.T.Wang

China, Vietnam, elevation unknown.

**Phu Tho Province**: Ha Ho District, Ao Chau, *V.X. Phuong 3483* (HN).

**Elatostema crispulihirtellum** (W.T.Wang) Y.H.Tseng & A.K.Monro, *comb. nov.*

Basionym: *Pellionia crispulihirtella* W.T.Wang, Bull. Bot. Res., Harbin 3(3): 57. 1983. Type: China. Yunnan, Pingbian, Shantou county, *C.W. Wang 82383* (holotype: PE!).

China, Vietnam; 710 m.

**Lao Cai Province**: On the way from Lao Cai to Sa Pa, *Sino-Vietnam Expedition 856* (PE).

**Elatostema cyrtandrifolium** (Zoll. & Mor.) Miq.

*Procris cyrtandrifolia* Zoll. & Mor.

*Elatostema herbaceifolium* Hayata, *E. sessile* var. *cyrtandrifolium* (Zoll. & Mor.) Wedd., *E. sessile* var. *pubescens* Hook.f., *E. herbaceifolium* Hayata.

Bhutan, China, India, Indonesia, Malaysia, Myanmar, Thailand, Vietnam; 200–1,050 m.

**Cao Bang Province**: Tra Linh District, Luu Ngoc Municipality, 22°47′N 106°18′E, *L. Averyanov et al. CBL1203* (LE, MO); **Dien Bien Province**: Tua Chua District, Xa Nhe Municipality, 21°52′35″N 103°24′21″E, *L. Averyanov et al. CPC2221* (LE); **Ha Giang Province**: between Meo Vac District and Dong Van District, 23°13′45″N 105°24′48″E, *L.F. Fu et al. VMN_CN244* (IBK, VNMN); Meo Vac District, Sung Cha Municipality, 23°11′N 105°17′E, *N.T. Hiep et al. NTH3302* (LE); **Hai Phong City**: Cat Hai District, Cat Ba Island, *N. Arnautov et al. LX-VN3796* (LE); **Ninh Binh Province**: Nho Quan District, Cuc Phuong National Park, *J.M. Hu Hu2187* (TAI); **Quang Binh Province**: Minh Hoa District, Thong Hoa Municipality, 17°40′N 105°57′E, *L. Averyanov et al. VH4793* (HN, LE, MO); **Son La Province**: Moc Chau District, Chieng Hac Municipality, 20°51′N 104°38′E, *N.T. Hiep et al. NTH2937* (LE); **Thanh Hoa Province**, Quy Chau District, Pu Huong Natural Reserve, *F. Wen et al. VMN_CN421* (IBK, VNMN).

**Elatostema dissectum** Wedd., Arch. Mus. Hist. Nat. 9(1–2): 314. 1856. Type: India, Khasia, *D. Hooker & T. Thomson s.n.* (lectotype: designated by Lin, Duan & Yang, 2009: 1910, P!).

*Elatostema paragungshanense* W.T.Wang, *Pellionia tonkinensis* Gagnep.

*Pellionia tetramera* Gagnep., Bull. Soc. Bot. France 75: 925. 1928, *syn. nov.* Type: Vietnam. Ba Vi District, Ba Vi Mountain, *B. Balansa 2536* (holotype: P!).

Bhutan, China, India, Laos, Sikkim, Thailand, Vietnam; 200–1,350 m.

**Bac Kan Province**: Phu Thong Hoa, *P.A. Eberharadt 4724* (P); **Ha Noi City**: Ba Vi District, Ba Vi National Park, *V.D. Nguyen et al. HNK 1268* (K); Ba Vi District, Ba Vi Mountain, *B. Balansa 2536* (P); **Ha Giang province**: Quan Ba District, Tung Vai Municipality, Thang village, 23°03′13″N 104°51′49″E, *L. Averyanov et al. VR554, VR559, VR563, VR660, VR713* (LE); **Hoa Binh Province**: Da Bac District, Cao Son Municipality, Buoi Truong Station, 20°52′31″N 105°11′48″E, *N.T. Hiep et al. HAL540* (HN, MO); Mai Chau District, *F. Wen et al. VMN_CN262* (IBK, VNMN); Mai Chou District, Hang Kia-Pa Co Nature Reserve, *F. Wen et al. VMN_CN326*, *VMN_CN331*, *VMN_CN337* (IBK, VNMN); **Kon Tum Province**: Dak Glei District, *L. Averyanov et al. VH1757* (HN, MO); **Lang Son Province**: Bac Son District, Tan Tri Municipality, *L. Averyanov et al. HAL6783* (LE); **Lao Cai Province**: Sa Pa District, Hoang Lien National Park, Ton Forest Station, *L.F. Fu et al. VMN_CN916* (IBK, VNMN); Van Ban District, Khanh Yen Ha Municipality, to the S of Na Nheo Village, 21°59′04″N 104°15′08″E, *L. Averyanov et al. HAL2814* (HN, LE); Van Ban District, Nam Xe Municipality, 22°01′27″N 104°00′25″E, *D.K. Harder et al. DKH7051* (LE); **Nghe An Province**: Con Cuong District, Chau Khe Municipality, Pu Mat National Park, 18°55′56″N 104°35′01″E, *M.S. Nuarliev 2128* (MW); Quy Chau District, Pu Huong Natural Reserve, *F. Wen et al. VMN_CN486* (IBK, VNMN); Tuong Duong District, Tam Hop Municipality, 19°05′07″N 104°20′55″E, *L. Averyanov et al. HLF7074* (LE); **Quang Binh Province**: Minh Hoa District, Dan Hoa Municipality, 17°41′50″N 105°45′54″E, *L. Averyanov et al. HAL12410* (LE); **Quang Tri Province**: Huong Hoa District, Huong Viet Municipality, 16°51′14″N 106°34′13″E, *N.T. Hiep et al. HLF5947* (LE); **Son La Province**: Chieng Yen District, *N.H. Hien et al. VN239* (HN); **Tuyen Quang Province**: Bach Ngoc, *P.A. Eberhardt 4818* (P); Na Hang District, Vinh Yen Municipality, 22°21′09″N 105°25′20″E, *L.V. Cham et al. HLF299* (HN).

We place *Pellionia tetramera* in synonymy based on its shared characters with *Elatostema dissectum*: oblong-lanceolate lamina and staminate inflorescence with long peduncle.

**Elatostema eberhardtii** (Gagnep.) Y.H.Tseng & A.K.Monro, *comb. nov.*

Basionym: *Pellionia eberhardtii* Gagnep., Bull. Soc. Bot. France 75: 921. 1928. Type: Vietnam. Quang Tri, Cu Bi, *P.A. Eberharadt 1962* (holotype: P!).

Endemic; elevation unknown.

**Quang Tri Province**: Cu Bi, *P.A. Eberharadt 1962* (P).

**Elatostema eurhynchum** Miq.

Indonesia, Vietnam; 1,600 m.

**Kon Tum Province**: Dak Glei District, 15°04′00″N 107°59′00″E, *L. Averyanov et al. VH797* (HN, LE, MO).

***Elatostema fengshanense** W.T.Wang & Y.G.Wei

China, Vietnam; elevation unknown.

**Cao Bang Province**: Ha Quang District, Pac Bo, *L.F. Fu et al. VMN_CN786* (IBK, VNMN).

**Elatostema ficoides** Wedd.

*Elatostema mariannae* C.B.Clarke.

China, India, Nepal, Sikkim, Vietnam; 400 m.

**Bac Kan Province**: Na Ri District, Kim Hy Nature Reserve, 22°11′56″N 106°04′04″E, *L.F. Fu et al. VMN_CN129* (IBK, VNMN). **Hoa Binh Province**: Crieng Xen, *P.A. Petelot 2965* (P).

***Elatostema glochidioides** W.T.Wang

China, Vietnam; 300–1,190 m.

**Ha Giang Province**: Between Meo Vac District and Dong Van District, *L.F. Fu et al. VMN_CN233* (IBK, VNMN); Quan Ba District, Bat Dai Son Nature Reserve, Thanh Van commune, Mo Sai village, 23°07′46″N 104°57′56″E, *L. Averyanov et al. VR417* (LE); Quan Ba District, Thang village, 23°03′13″N 104°51′49″E *L. Averyanov et al. VR553* (LE).

**Elatostema grijsii** (Hance) Y.H.Tseng & A.K.Monro, *comb. nov.*

Basionym: *Pellionia grijsii* Hance, J. Bot. 6(62): 49. 1868. Type: China. Fujian, *Exsicc. 6704* (holotype: BM!).

*Pellionia funingensis* W.T.Wang*.*

China, Vietnam; 800–1,900 m.

**Cao Bang Province**: Nguyen Binh District, Nguyen Binh Municipality, 22°37′N 105°52′E, *L. Averyanov et al. CBL069* (LE, MO); Nguyen Binh District, Phia Oac—Phia Den Nature Reserve, 22°36′24″N 105°52′46″E, *L.F. Fu et al. VMN_CN157* (IBK, VNMN); **Lang Son Province**: Bac Son District, Tan Tri Municipality, *L. Averyanov et al. HAL6753* (LE); **Lao Cai Province**: Van Ban District, Hoang Lien - Van Ban Nature Reserve, *L.F. Fu et al. VMN_CN921* (IBK, VNMN); **Vinh Phuc Province**: Tam Dao Range, *N. Arnautov et al. LX-VN4004* (LE).

**Elatostema heterolobum** (Wedd.) Hallier f.

*Pellionia heteroloba* Wedd.

*Elatostema bicuspidatum* Hallier f., *E. griffithianum* (Wedd.) Hallier f., *E. henryanum* Hand.-Mazz., *Pellionia heteroloba* var. *minor* W.T.Wang, *P. keitaoensis* Yamam., *P.  menglianensis* Y.Y.Qian.

Bhutan, China, India, Laos, Myanmar, Sikkim, Vietnam; 400–1,900 m.

**Cao Bang Province**: Bao Lac District, Dinh Phung Municipality, *L.F. Fu et al. VMN_CN816* (IBK, VNMN); Nguyen Binh District, Nguyen Binh Municipality, 22°37′N 105°52′E, *L. Averyanov et al. CBL035, CBL062* (HN, LE, MO); **Ha Giang Province**: Meo Vac District, Vicinities of Meo Vac Town, 23°10′N 105°24′E, *N.T. Hiep et al. NTH3385* (LE); Quan Ba District, Tung Vai Municipality, *L.F. Fu et al. VMN_CN870* (IBK, VNMN); **Ha Noi City**: Ba Vi District, Ba Vi National Park, *V.D. Nguyen et al. HNK1266* (K); **Hoa Binh Province**: Mai Chau District, Pa Co Municipality, *V.X. Phuong 2397* (HN); **Lao Cai Province**: Sa Pa District, *B. Khao & V. Trung 2545* (HN); Van Ban District, Khanh Yen Ha Municipality, 21°58′28″N 104°15′52″E, *L. Averyanov et al. HAL2312* (LE, MO); **Nghe An Province**: Con Cuong District, Chau Khe Municipality, Pu Mat National Park, 18°55′51″N 104°36′20″E, *M.S. Nuarliev et al. 2042* (MW); **Quang Binh Province**: Minh Hoa District, Dan Hoa Municipality, 17°41′50″N 105° 45′54″E, *L. Averyanov et al. HAL12250, HAL12412* (LE); **Son La Province**: Van Ho District, Tan Xuan Municipality, 20°40′33″N 104°39′00″E, *L. Averyanov et al. CPC7127* (LE).

**Elatostema heyneanum** (Wedd.) Hallier f.

*Pellionia heyneana* Wedd.

Cambodia, China, India, Indonesia, Sri Lanka, Thailand, Vietnam; 100–300 m.

**Ha Noi City:** Ba Vi District, Ba Vi Mountain, *B. Balansa 2549* (P); **Quang Binh Province**: Tuyen Hoa District, Lam Hoa Municipality, 17°56′51″N 105°49′18″E, *L. Averyanov et al. CPC2679, CPC2696* (LE); **Quang Tri Province**: Da Krong District, Ta Rut Municipality, 16°24′29″N 107°01′07″E, *L. Averyanov et al. CPC3049* (LE).

**Elatostema hookerianum** Wedd., Arch. Mus. Hist. Nat. 9(1–2): 309. 1856, Type: Sikkim, *J.D. Hooker s.n.* (holotype: P!, isotype: MPU!).

*Elatostema subfalcatum* W.T.Wang*.*

*Pellionia macroceras* Gagnep., Bull. Soc. Bot. France 75: 922. 1928, *syn. nov.* Type: Vietnam. Lao Cai, Sa Pa District, *P.A. Petelot 2294* (lectotype: P: P00601742!, designated here, isolectotype: P: P00601741!).

Bhutan, China, India, Sikkim, Vietnam; 1,200–2,100 m.

**Lai Chau Province**: Than Uyen District, Ho Mit Municipality, 22°06′N 103°52′E, *N.T. Hiep et al. NTH2668, NTH2809, NTH2832, NTH2874* (LE); **Lao Cai Province**: Sa Pa District, Hoang Lien National Park, *L.F. Fu et al. VMN_CN882* (IBK, VNMN), *P.A. Petelot 2294* (P); **Son La Province**: Yen Chau District, Muong Lum Municipality, Lum Village, 21°00′17″N 104°29′08″E, *D.K. Harder et al. 7217* (HN); **Province unknown** (Northern Vietnam): *G.V. Mikeshin s.n.* (MHA).

Note: The protologue of *Pellionia macroceras* (Gagnepain, 1928) includes a description of the species with two type localities of ‘Chapa’ (Vietnam) and Sikkim. The former relates to the collection *P.A. Petelot 2294* with two duplicates housed at P (barcodes P00601741, P00601742), while the latter is represented by the collection *J. de Rémy 1863* with a single specimen at P (barcode P00601743). All of these specimens contain the name ‘*Pellionia macroceras* Gagnep.’ written in Gagnapain’s handwriting and match the protologue description well. The dates of these collections (1925 and 1863) are before the publication of the protologue (1928), and therefore were used during its preparation. We select the specimen *P.A. Petelot 2294* (P: P00601742) as the lectotype because it contains anatomical drawing with short notes made by Gagnepain; the other one (P: P00601741) is isolectotype. The status of the specimen *J. de Rémy 1863* (P: P00601743) can be indicated as a remaining syntype.

We place *Pellionia macroceras* in synonymy based on the same characters of falcate lamina with cordate base with *Elatostema hookerianum*.

**Elatostema integrifolium** (D.Don) Wedd.

*Procris integrifolia* D.Don.

*Elatostema cuspidiferum* Miq., *E. miquelianum* Wedd., *E. pulgarense* Elmer, *E. sesquifolium* (Reinw. ex Blume) Hassk., *E. sesquifolium* var. *integrifolium* (D.Don) Wedd., *E. viridicaule* W.T.Wang, *E. zollingerianum* Wedd., *Procris sesquifolia* Reinw. ex Blume.

Bhutan, China, India, Indonesia, Myanmar, Nepal, Thailand, Vietnam; 600–900 m.

**Cao Bang Province**: Bao Lac District, Dinh Phung Municipality, *L.F. Fu et al. VMN_CN819* (IBK, VNMN); **Hai Phong City**: Cat Hai District, *Y.G. Wei et al. VMN_CN06* (IBK, VNMN); Cat Hai District, Cat Ba Island, *N. Arnautov et al. LX-VN3778* (LE); **Hoa Binh Province**: Lac Son District, Tu Do Municipality, 20°25′29″N 105°19′36″E, *N.Q. Hieu et al. CPC1538* (LE); **Kon Tum Province**: Sa Thay District, Ro Koi Municipality, Chu Mom Ray National Park, 14°28′20″N 107°43′00″E, *M.S. Nuraliev & S.P. Kuznetsova 1257* (IBK, MW); **Lam Dong Province**: Bao Loc District, *L. Averyanov et al. III-LX-VN1183* (LE); **Quang Tri Province**: Da Krong District, Ta Rut Municipality, 16°24′29″N 107°01′07″E, *L. Averyanov et al. CPC3039* (LE); Huong Hoa District, Huong Viet Municipality, 16°51′06″N 106°34′38″E, *L. Averyanov et al. CPC2815* (LE).

**Elatostema kimhyense** Y.G.Wei & V.T.Do

Endemic; 350–1,200 m.

**Bac Kan Province**: Na Ri District, Kim Hy National Park, 22°11′56″N 106°04′05″E, *Y.G. Wei & V.T. Do V-23* (IBK, PE, VNMN), *L.F. Fu et al. VMN_CN194* (IBK, VNMN).

**Elatostema laevissimum** W.T.Wang

*Elatostema laevissimum* W.T.Wang var. *puberulum* W.T.Wang.

China, Vietnam; 780–2,200 m.

**Ha Giang Province**: Meo Vac District, Vicinities of Meo Vac Town, 23°10′N 105°24′E, *N.T. Hiep et al. NTH3378, NTH3390* (LE); Quan Ba District, Tung Vai, *L.F. Fu et al. VMN_CN871* (IBK, VNMN); between Meo Vac District and Dong Van District, 23°07′31″N 105°25′42″E, *L.F. Fu et al. VMN_CN188* (IBK, VNMN); **Kon Tum Province**: Dak Glei District, 15°04′23″N 108°01′34″E, *L. Averyanov et al. VH062* (HN, MO), 15°04′00″N 107°59′00″E, *L. Averyanov et al. VH629* (HN, LE, MO), *VH074, VH590* (LE, MO); **Lai Chau Province**: Than Uyen District, Ho Mit Municipality, 22°06′N 103°52′E, *N.T. Hiep et al. NTH2812* (LE); **Lao Cai Province**: Van Ban District, 21°58′42″N 104°00′37″E, *L. Averyanov et al. HAL2013* (LE); **Nghe An Province:** Que Phong District, Hanh Dich Municipality, Pu Hoat Nature Reserve, 19°44′15″N 104°48′59″E, *M.S. Nuraliev 2192* (MW); **Quang Nam Province**: *L. Averyanov et al. VH923* (LE).

**Elatostema leiocarpum** (W.T.Wang) Y.H.Tseng & A.K.Monro, *comb. nov.*

Basionym: *Pellionia leiocarpa* W.T.Wang, Guihaia 2(3): 115. 1982. Type: China. Guangxi, Napo, Baidu, Nunghua, *D. Fang et al. 25142* (holotype: GXMI!).

China, Vietnam; ca. 1,200 m.

**Ha Giang Province**: Quan Ba District, Bat Dai Son Nature Reserve, 23°09′18″N 105°00′09″E, *D.K. Harder et al. 5029* (HN); Quan Ba District, Can Ty Municipality, 23°04′N 104°59′E, *N.T. Hiep et al. NTH3618* (LE); Quan Ba District, San Chu village, 23°08′55″N 104°59′46″E, *L. Averyanov et al. VR079* (LE); Quan Ba District, Tung Vai Municipality, Thang village, 23°03′41″N 104°50′42″E, *L. Averyanov et al. VR575* (LE).

**Elatostema lineolatum** Wight

*Elatostema lineolatum* var. *majus* Wedd., *E. tumidulum* Koidz.

Bhutan, China, India, Myanmar, Nepal, Sikkim, Sri Lanka, Thailand; 150–1,300 m.

**Cao Bang Province**: Bao Lac District, Xuan Truong Municipality, *L.F. Fu et al. VMN_CN809* (IBK, VNMN); Nguyen Binh District, Phia Oac—Phia Den Nature Reserve, 22°37′40″N 105°53′10″E, *L.F. Fu et al. VMN_CN163* (IBK, VNMN); **Dak Lak Province**: Lak District, Bong Krang Municipality, Chu Yang Sin National Park, 12°25′00″N 108°23′00″E, *M.S. Nuraliev et al. 925* (K, MW); **Ha Giang Province**: Bac Me District, Bac Me Nature Reserve, *L.F. Fu et al. VMN_CN232* (IBK, VNMN); **Gia Lai Province**: K’Bang District, Son Lang Municipality, Kon Chu Rang Nature Reserve, 14°30′55″N 108°32′48″E, *M.S. Nuraliev 2010* (MW), 14°30′59″N 108°32′27″E, *M.S. Nuraliev 2020* (MW); **Lang Son Province**: Bac Son District, Tan Tri Municipality, *L. Averyanov et al. HAL6787* (LE); **Lao Cai Province**: Sa Pa District, San Sa Ho Municipality, Hoang lien National Park, Cat Cat village area, 22°20′N 103°49′E, *M.S. Nuraliev & D.D. Sokoloff 282* (MW); Van Ban District, Nam Xe Municipality, 22°02′35″N 103°57′23″E, *D.K. Harder et al. 7010* (HN, LE); **Nghe An Province**: Con Cuong District, Chau Khe Municipality, Pu Mat National Park, 18°57′38″N 104°40′44″E, *M.S. Nuraliev 2065* (MW); Tuong Duong District, Tam Quang Municipality, 19°03′09″N 104°36′53″E, *L. Averyanov et al. HLF6758* (LE); **Quang Binh Province**: Bo Trach District, Hung Trach Municipality, 17°27′30″N 106°23′06″E, *S.K. Wu et al. WP923* (HN); **Vinh Phuc Province**: *N. Arnautov et al. LX-VN3985* (LE); Tam Dao District, 21°28′02″N 105°38′39″E, *L.F. Fu et al. VMN_CN51* (IBK, VNMN).

**Elatostema longibracteatum** W.T.Wang

a. **Elatostema longibracteatum** var. **glabricaule** W.T.Wang

Endemic; 650–800 m.

**Cao Bang Province**: Tra Linh District, Quoc Toan Municipality, *L. Averyanov et al. VH2468* (HN, LE); 22°45′25″N 106°17′58″E, *D.K. Harder et al. 4288* (K); Tra Linh District, Luu Ngoc Municipality, 22°47′N 106°18′E, *L. Averyanov et al. CBL1199* (LE, MO).

This variety is very similar to *Elatostema longibracteatum* W.T.Wang var. *longibracteatum* and further study may result in it being treated as a synonym.

**Elatostema longipedunculatum** (W.T.Wang) Y.H.Tseng & A.K.Monro, *comb. nov.*

Basionym: *Pellionia longipedunculata* W.T.Wang, Bull. Bot. Res., Harbin 2(1): 1. 1982. Type: China. Guangxi, Shangsi County, Shiwandashan, *S.C. Chen 4918* (holotype: IBSC!).

*Pellionia subundulata* W.T.Wang var. *angustifolia* W.T.Wang*.*

China, Vietnam; 197–1,000 m.

**Ha Noi City**: Ba Vi District, Ba Vi Mountain, 21°04.096′N 105°21.501′E, *N.A. Vislobokov & S.P. Kuznetsova 681* (MW); **Ha Tinh Province**: Huong Son District, Rao An-Ngam Thep village, 18°21′53″N 105°13′12″E, *T.H. Nguyen et al. 184* (MO); **Vinh Phuc Province**: Tam Dao District, *Sino-Vietnam Expedition 1996A* (PE).

**Elatostema longistipulum** Hand.-Mazz.

China, Vietnam; 140–1,450 m.

**Cao Bang Province**: Ha Lang District, Thang Loi Municipality, 22°45′N 106°42′E, *P.K. Loc et al. CBL1661* (LE, MO); **Ha Giang Province**: Bac Me District, Bac Me Nature Reserve, 22°38′37″N 105°28′36″E, *L.F. Fu et al. VMN_CN220* (IBK, VNMN); **Ha Noi City**: Ba Vi District, Ba Vi National Park, *V.D. Nguyen et al. HNK1282* (K); **Hoa Binh Province**: Lac Son District, Tu Do Municipality, 20°24′57″N 105°13′16″E, *N.Q. Hieu et al. CPC1403* (LE); Mai Chau District, *F. Wen et al. VMN_CN274, VMN_CN317, VMN_CN323* (IBK, VNMN); **Lang Son Province**: Huu Lung District, Huu Lien Municipality, 21°40′59″N 106°20′30″E, *D.K. Harder 4116* (MO); **Nghe An Province**: Con Cuong District, Chau Khe Municipality, Pu Mat National Park, 18°57′42″N 104°41′40″E, *M.S. Nuraliev 2077* (MW); Quy Hop District, Chau Thai Municipality, Bom Village, *V.D. Nguyen et al. HNK1611* (K); **Ninh Binh Province**: Nho Quan District, Cuc Phuong National Park, *J.M. Hu Hu2110* (TAI); *L. Averyanov et al. LX-VN1732* (LE), *V.D. Nguyen et al. HNK1334* (K); **Phu Tho Province**: Tan Son District, Xuan Son Municipality, 21°02′55″N 104°56′51″E, *L. Averyanov et al. HAL12722* (LE), 21°06′57″N 104°57′17″E, *L. Averyanov et al. HAL12677* (LE); **Quang Binh Province**: *G.V. Mikeshin, O.V. Daeva s.n.* (MHA); **Son La Province**: Van Ho District, Tan Xuan Municipality, 20°40′33″N 104°39′00″E, *L. Averyanov et al. CPC7143* (LE); Yen Chau District, Muong Lum Municipality, 21°00′35″N 104°29′06″E, *L. Averyanov et al. CPC1884* (LE); **Thai Nguyen Province**: Vo Nhai District, Than Sa Municipality, 21°48′N 105°54′E, *N.T. Hiep et al. NTH3780* (LE); **Thanh Hoa Province**: Quy Chau District, Pu Huong Natural Reserve, *F. Wen et al. VMN_CN428* (IBK, VNMN); Quan Hoa District, Hien Chung Municipality, Xuan Lien Reserve, *F. Wen et al. VMN_CN398, VMN_CN410* (IBK, VNMN); Quan Hoa District, Pu Hu Nature Reserve; *F. Wen et al. VMN_CN350* (IBK, VNMN); Thuong Xuan District, Bat Mot Municipality, 19°59′11″N 104°58′32″E, *L. Averyanov et al. CPC6440* (LE); **Tuyen Quang Province**: Na Hang District, Vinh Yen Municipality, 22°21′14″N 105°25′11″E, *P.K. Loc et al. HLF-009* (HN).

**Elatostema macintyrei** Dunn

Bhutan, China, India, Thailand, Vietnam; 1,000–1,100 m.

**Cao Bang Province**: Bao Lac District, Xuan Truong Municipality, *L.F. Fu et al. VMN_CN807* (IBK, VNMN); **Gia Lai Province**: Mang Yang District, A Yun Municipality, Kon Ka Kinh National Park, 14°13′17″N 108°18′59″E, *M.S. Nuraliev et al. 1492* (IBK, MW), *M.S. Nuraliev 1882* (IBK, MW); **Ha Giang Province**: between Meo Vac District and Dong Van District, 23°09′06″N 105°24′59″E, *L.F. Fu et al. VMN_CN185* (IBK, VNMN).

***Elatostema malacotrichum** W.T.Wang & Y.G.Wei

China, Vietnam; 400 m.

**Cao Bang Province**: Ha Lang District, An Lac Municipality, 22°46′N 103°44′E, *L. Averyanov et al. CBL607* (LE); Ha Quang District, Pac Bo, *L.F. Fu et al. VMN_CN784* (IBK, VNMN).

** Elatostema monandrum** (Buch.-Ham. ex D.Don) H.Hara

*Procris monandra* Buch.-Ham. ex D.Don.

*Elatostema diversifolium* Wedd., *E. laetum* Wedd., *E. monandrum* var. *ciliatum* (Hook.f.) Murti, *E. monandrum* var. *pinnatifidum* (Hook.f.) Murti, *E. monandrum* var. *serpens* (Hook.f.) Murti, *E. monandrum* var. *zeylanicum* (Hook.f.) Murti, *E. muscicola* W.T.Wang, *E. surculosum* Wight, *Pellionia mairei* H.Lév.

Bhutan, China, India, Myanmar, Nepal, Sikkim, Sri Lanka, Thailand, Vietnam; 2,000–2,300 m.

**Lao Cai Province**: Sa Pa District, *P.A. Petelot 5049* (P); **Nghe An Province**: Ky Son District, Na Ngoi Municipality, 19°11′58″N 104°11′39″E, *L. Averyanov et al. CPC6150* (LE); 19°12′23″N 104°11′34″E, *L. Averyanov et al. CPC6215* (LE).

**Elatostema myrtillus** (H.Lév.) Hand.-Mazz., Symb. Sin. 7(1): 146. 1929. Types: China. Pin-Fa, *J. Cavalerie 559* (syntype: P!); Kouy-Tchéou, *E. Bodinier 1548* (syntype: E); Kan-Yen-Tong, *J. Esquirol 698* (syntype: E).

*Pellionia myrtillus* H.Lév.

*Elatostema colaniae* Gagnep., Bull. Soc. Bot. France 76: 81. 1929, *syn. nov.* Type: Vietnam. Lang Met, *P.A. Colani 2927* (holotype: P!, isotype: P!).

China, Vietnam; 500–1,600 m.

**Bac Giang Province**: Lang Met, *P.A. Colani 2927* (P); **Cao Bang Province**: Tra Linh District, Quoc Toan Municipality, vicinity of Thang Heng and Lung Tao Villages, 22°45′30″N 106°17′22″E, *L. Averyanov et al. VH4855* (HN, LE); Tra Linh District, Quoc Toan Municipality, 22°45′15″N 106°17′54″E, *D.K. Harder et al. 4312* (K); **Ha Giang Province**: between Meo Vac District and Dong Van District, 23°15′02″N 105°23′09″E, *L.F. Fu et al. VMN_CN197* (IBK, VNMN); Meo Vac District, Sung Cha Municipality, 23°11′N 105°17′E, *N.T. Hiep et al. NTH3287* (K, LE); Meo Vac District, Sung Cha Municipality, 23°10′12″N 105°17′39″E, *L. Averyanov et al. HAL8490* (LE); Quan Ba District, Bat Dai Son Nature Reserve, Thanh Van commune, Mo Sai village, 23°07′46″N 104°57′56″E, *L. Averyanov et al. VR409* (LE); Quan Ba District, Tung Vai Municipality, *L.F. Fu et al. VMN_CN869* (IBK, VNMN); Quan Ba District, Tung Vai Municipality, Thang village, 23°03′13″N 104°51′49″E, *L. Averyanov et al. VR742* (LE).

We place *Elatostema colaniae* in synonymy based on the same combination of characters as in *E. myrtillus*: small lamina (13–28 × 6–10 mm) with auriculate base and furfuraceous stem base.

***Elatostema nanchuanense** W.T.Wang

China, Vietnam; 1,050–1,150 m.

**Ha Giang province**: Quan Ba District, Tung Vai Municipality, Thang village, 23°03′13″N 104°51′49″E, *L. Averyanov et al. VR552* (LE).

**Elatostema nasutum** Hook.f.

*Elatostema hainanense* W.T.Wang, *E. stipulosum* Hand.-Mazz.

Bhutan, China, Nepal, Vietnam; 1,100–2,100 m.

**Cao Bang Province**: Nguyen Binh District, Nguyen Binh Municipality, 22°37′N 105°52′E, *L. Averyanov et al. CBL077, CBL171* (LE, MO); Nguyen Binh District, Phia Oac–Phia Den Nature Reserve, 22°36′29″N 105°52′18″E, *L.F. Fu et al. VMN_CN162* (IBK, VNMN); **Ha Giang Province**: Vi Xuyen District, Cao Bo Municipality, Tam Ve village, 22°46′30″N 104°49′01″E, *D.K. Harder 5425* (MO); Yen Minh District, Du Gia Municipality, [23°56′N 105°13″E], *P.K. Loc et al. CBL2028, CBL2080* (LE, MO); **Kon Tum Province**: Dak Glei District, *N.T. Hiep et al. VH1757* (LE); **Lai Chau Province**: Than Uyen District, Ho Mit Municipality, 22°06′N 103°52′E, *N.T. Hiep et al. NTH2669, NTH2812, NTH2874* (LE); **Lao Cai Province**: Sa Pa District, San Sa Ho, Sin Chai Municipality, 22°20′44.3″N 103°48′77.0″E, *V.X. Phuong et al. HNK470* (K); **Nghe An Province**: Ky Son District, Na Ngoi Municipality, 19°11′59″N 104°11′24″E, *N.T. Hiep et al. CPC6286* (LE); **Son La Province**: Bac Yen District, Chu Village, 21°15′40.0″N 104°29′27.0″E, *V.D. Nguyen et al. HNK2726* (K); **Thanh Hoa Province**: Thuong Xuan District, Bat Mot Municipality, 19°58′18″N 104°59′24″E, *L. Averyanov et al. CPC6555* (LE); **Vinh Phuc Province**: Tam Dao District, Tam Dao Range, *N. Arnautov et al. LX-VN3858* (LE).

**Elatostema neobaviense** Y.H.Tseng & A.K.Monro *nom. nov.*

non *E. baviense* Gagnep.

*Pellionia baviensis* Gagnep., Bull. Soc. Bot. France 75: 919. 1928. Type: Vietnam. Ha Noi City, Ba Vi District, Ba Vi Mountain, *B. Balansa 2548* (lectotype: designated by Lin & Yang, 2010: 1912, P!).

Endemic; elevation unknown.

**Ha Noi City**: Ba Vi District, Ba Vi Mountain, *B. Balansa 2548* (P).

We select ‘*neobaviense*’ as epiphet as it takes the place of ‘*baviense*’ which would be in conflict with the homonym *Elatostema baviensis*.

**Elatostema neriifolium** W.T.Wang & Z.Y.Wu

China, Vietnam; 50–750 m.

**Lao Cai Province**: *Sino-Vietnam Expedition 755* (PE); **Quang Binh Province**: Minh Hoa District, Thuong Hoa Municipality, 17°39′22″N 105°54′59″E, *N.T. Hiep et al. CPC3819* (LE); **Thanh Hoa Province**: Ba Thuoc District, Co Lung Municipality, Khuyn village, 20°26′39″N 105°15′33″E, *L. Averyanov et al. 3130* (MO); 10 km SW of Ba Thuoc, *B.A. Korotiaev s.n.* (LE); **Tuyen Quang Province**: Na Hang District, Thanh Tuong Municipality, 22°16′26″N 105°25′58″E, *P.K. Loc et al. HAL149* (HN).

***Elatostema oblongifolium** S.H.Fu

*Pellionia bodinieri* H.Lév.

*Elatostema multicanaliculatum* B.L.Shih & Yuen P.Yang.

China, Vietnam; 1,050–1,400 m.

**Ha Giang province**: Quan Ba District, Tung Vai Municipality, Thang village, 23°03′13″N 104°51′49″E, *L. Averyanov et al. VR560* (LE), 23°03′42″N 104°50′42″E, *L. Averyanov et al. VR634* (LE).

***Elatostema obtusum** Wedd.

*Dorstenia pubescens* Blanco, *Elatostema delicatulum* Wedd.

Bhutan, China, India, Nepal, Sikkim, Thailand, Vietnam; 1,400–1,900 m.

**Cao Bang Province**: Nguyen Binh District, Nguyen Binh Municipality, 22°37′N 105°52′E, *L. Averyanov et al. CBL206* (LE, MO, P); **Lao Cai Province**: Sa Pa District, Hoang Lien National Park, Ton Forest Station, *L.F. Fu et al. VMN_CN971* (IBK, VNMN).

***Elatostema oppositum** Q.Lin & Y.M.Shui

China, Vietnam; ca. 1,100 m.

**Ha Giang Province**: Quan Ba District, 23°06′57″N 105°01′48″E, *D.K. Harder et al. 4973* (HN, MO), *4997* (HN).

**Elatostema paucidentatum** H.Schroet.

a. **Elatostema paucidentatum** var. **paucidentatum**

*Pellionia paucidentata* (H.Schroet.) Chien.

*Elatostema henryanum* var. *oligodontum* Hand.-Mazz., *Pellionia longgangensis* W.T.Wang, *P. paucidentata* var. *hainanica* Chien & S.H.Wu, *P. subundulata* W.T.Wang.

China, Vietnam; 180–300 m.

**Cao Bang Province**: Bao Lam District, Bac Mieu Municipality, *L.F. Fu et al. VMN_CN827* (IBK, VNMN); **Ha Giang Province**: between Meo Vac District and Dong Van District, *L.F. Fu et al. VMN_CN240* (IBK, VNMN); **Lai Chau Province**: Muong Te District, Muong Te Municipality, 22°32′06″N 102°32′29″E, *N.T. Hiep et al. HAL10102, HAL10123* (LE); **Lang Son Province**: Lung District, Huu Lien Municipality, 21°39′31″N 106°22′06″E, *D.E. Atha et al. NY-HN 73* (MO); **Ninh Binh Province**: Nho Quan District, Cuc Phuong National Park, 20°17′33″N 105°39′54″E, *D.D. Soejarto 10498* (MO), 20°17.55′N 105°39.90′E, *N.T. Hiep et al. 1998* (P); **Quang Ninh province**: Dam Ha District, Lomg Ngong village, Sai Vong Mo Leng mt., *W.T. Tsang 30277* (SING); Ha-coi, near Chuk-phai, Taai Wong Mo Shan, *W.T. Tsang 29166* (SING); **Thai Nguyen Province**: Vo Nhai District, Thuong Lung Municipality, 21°48.75′N 105°58.09′E, *L.Q. Li et al. 0078* (HN); **Thanh Hoa Province**: Quan Hoa District, Hien Chung Municipality, Xuan Lien Nature Reserve, *F. Wen et al. VMN_CN413* (IBK, VNMN); **Vinh Phuc Province**: *N. Arnautov et al. LX-VN3988* (LE).

b. **Elatostema paucidentatum** var. **dolichoceras** W.T.Wang

Endemic; 100–150 m.

**Lao Cai Province**: *Sino-Vietnam Expedition 590* (PE).

***Elatostema pergameneum** W.T.Wang

China, Vietnam; 100–250 m.

**Lang Son Province**: Huu Lung District, Huu Lien Municipality, 21°40′59″N 106°20′30″E, *D.K. Harder et al. 4121* (K); **Quan Binh Province**: Minh Hoa District, 17°40′N 105°57′E, *L. Averyanov et al. VH4611* (HN, LE).

**Elatostema petelotii** Gagnep.

China, Vietnam; 260–1,300 m.

**Cao Bang Province**: Phia Oac, *P.A. Petelot 720* (P); **Ha Giang Province**: between Meo Vac District and Dong Van District, *L.F. Fu et al. VMN_CN239* (IBK, VNMN); Quan Ba District, Tung Vai Municipality, *L.F. Fu et al. VMN_CN868* (IBK, VNMN); Quan Ba District, Thang village, around point 23°03′13″N 104°51′49″E, *L. Averyanov et al. VR540*, *VR731* (LE); Yen Minh District, Du Gia Municipality, 23°56′N 105°13′E, *P.K. Loc et al. CBL2022, CBL2024* (LE); **Nghe An Province**: Que Phong District, Hanh Dich Municipality, Pu Hoat Nature Reserve, 19°45′19″N 104°47′45″E, *M.S. Nuraliev 2142* (MW); **Phu Tho Province**: Thanh Son District, Xuan Son Municipality, *V.X. Phuong 3930* (HN).

**Elatostema platyphyllum** Wedd., Arch. Mus. Hist. Nat. 9: 301. 1856. Type: India. Khasia, *J.D. Hooker & T. Thomoson s.n.* (lectotype: designated by Lin, Duan & Yang, 2009: 1912, K!).

*E. platyphylloides* B.L.Shih & Yuen P.Yang, *E. platyphyllum* Wedd. var. *polycephalum* Hara*.*

*Elatostema baviensis* Gagnep., Bull. Soc. Bot. France 76: 80. 1929, *syn. nov.* Type: Vietnam. Ha Noi City, Ba Vi District, Ba Vi Mountain, *B. Balansa 2537* (holotype: P!).

Bhutan, China, India, Nepal, Philippines, Vietnam; 800–1,669 m.

**Gia Lai Province**: K’Bang District, Song Lang Municipality, Kon Chu Rang Nature Reserve, 14°31′04″N 108°36′23″E, *M.S. Nuraliev et al. 1996* (MW); **Ha Giang Province**: between Meo Vac District and Dong Van District, 23°13′44″N 105°24′48″E, *L.F. Fu et al. VMN_CN191* (IBK, VNMN); **Ha Noi City**: Ba Vi District, Ba Vi National Park, 21°04′23″N 105°21′36″E, *L.F. Fu et al. VMN_CN33* (IBK, VNMN), Ba Vi District, Ba Vi Mountain, *B. Balansa 2537* (P); **Ninh Binh Province**: Nho Quan District, Cuc Phuong National Park, *J.M. Hu Hu2122* (TAI); **Thanh Hoa Province**: Quan Hoa District, Hien Chung Municipality, Xuan Lien Nature Reserve, *F. Wen et al. VMN_CN399*, *VMN_CN408* (IBK, VNMN).

We place *Elatostema baviensis* in synonymy based on the same combination of characters as in *E. platyphyllum*: large lamina (10–25 × 4–7.5 cm) with auriculate base, large stipule (15–25 × 3–5.5 mm) and pistillate inflorescence with short peduncle and obvious receptacle.

***Elatostema prunifolium** W.T.Wang

China, Vietnam; elevation unknown.

**Ha Giang Province**: Quan Ba District, Tung Vai Municipality, *L.F. Fu et al. VMN_CN866* (IBK, VNMN).

**Elatostema pseudodissectum** W.T.Wang

China, Vietnam; 1,000–1,300 m.

**Cao Bang Province**: Bao Lac District, Ca Thanh Municipality, 22°44′N 105°50′E, *L.  Averyanov et al. CBL389* (LE, MO); **Vinh Phuc Province**: Tam Dao District, Tam Dao National Park, 21°27′47″N 105°38′68″E, *L.Q. Li et al. 559* (PE).

***Elatostema pseudolongipes** W.T.Wang & Y.G.Wei

China, Vietnam; 150–600 m.

**Bac Kan Province**: Ba Be District, Nam Mau Municipality, Ba Be National Park, 22°26.875′N 106°37.028′E, *D.E. Atha et al. DA4722* (HN); Na Ri District, Liem Thuy Municipality, 21°57.36′N 106°04.622′E, *D.E. Atha et al. DA4773* (HN), Na Ri District, Kim Hy Municipality, 22°17′N 106°03′E, *N.T. Hiep et al. NTH3688, NTH3691* (LE); **Cao Bang Province**: Tra Linh District, Quoc Toan Municipality, Thang Heng vicinity and Lung Tao Villages, 22°45′30″N 106°17′22″E, *L. Averyanov et al. VH4850, VH4928, VH4929* (HN, LE, MO); **Ha Giang Province**: Bac Me District, Bac Me Nature Reserve, 22°45′23″N 105°13′46″E, *L.F. Fu et al. VMN_CN221* (IBK, VNMN); **Ninh Binh Province**: Cuc Phuong National Park, *L. Wu et al. VN0049* (HN, IBK); **Quang Binh Province**: Bo Trach District, Son Trach Municipality, 17°31′02″N 106°16′48″E, *L. Averyanov et al. HAL6382* (LE); Minh Hoa District, Thuong Hoa Municipality, 17°39′12″N 105°54′53″E, *N.T. Hiep et al. CPC3753* (LE); Tuyen Hoa District, Lam Hoa Municipality, 17°56′10″N 105°49′15″E, *L. Averyanov et al. CPC2452* (LE); **Thanh Hoa Province**: Ba Thuoc District, Co Lung Municipality, Eo Dieu Village, 22°25′35″N 105°14′26″E, *L. Averyanov et al. HAL3354* (HN).

*** Elatostema** **pycnodontum** W.T.Wang

China, Vietnam; 1,400–1,500 m.

**Cao Bang Province**: Bao Lac District, Dinh Phuong Municipality, 22°47′N 105°49′E, *P.K. Loc et al. CBL1489* (LE, MO).

**Elatostema radicans** (Siebold & Zucc.) Wedd.

*Procris radicans* Siebold & Zucc.

*Elatostema cavaleriei* (H.Lév.) Hand.-Mazz., *E. radicans* var. *grande* (Gagnep.) H.Schroet., *Pellionia arisanensis* Hayata, *P. cavaleriei* H.Lév., *P. chikushiensis* Yamam., *P. iseana* (Makino ex Hatus.) Hatus. ex Kimura, *P. pauciflora* W.T.Wang, *P. radicans* (Siebold & Zucc.) Wedd., *P. radicans* f. *grandis* Gagnep., *P. radicans* var. *chikushiensis* (Yamam.) S.S.Ying, *P. radicans* var. *grandis* (Gagnep.) W.T.Wang*.*

China, Japan, Korea, Vietnam; 600–2,700 m.

**Cao Bang Province**: Nguyen Binh District, Nguyen Binh Municipality, 22°37′N 105°52′E, *L. Averyanov et al. CBL064* (HN, LE, MO); Nguyen Binh District, Phia Oac—Phia Den Nature Reserve, 22°36′29″N 105°52′18″E, *L.F. Fu et al. VMN_CN160* (IBK, VNMN); **Gia Lai Province**: K’Bang District, Son Lang Municipality, Kon Chu Rang Nature Reserve, 14°31′04″N 108°36′23″E, *M.S. Nuraliev et al. 1994* (MW), 14°30′59″N 108°32′27″E, *M.S. Nuraliev et al. 2019* (MW), 14°30′02″N 108°34′27″E, *M.S. Nuraliev et al. 2027* (MW); **Ha Giang Province**: Meo Vac District, vicinities of Meo Vac Town, 23°10′N 105°24′E, *N.T. Hiep et al. NTH3377* (LE); **Khanh Hoa Province**: Khanh Son District, 12°12′N 108°44′E, *L. Averyanov et al. VH4353* (LE, MO); **Kon Tum Province**: *L. Averyanov et al. VH543* (LE, MO); **Lam Dong Province**: Lac Duong District, Da Chais Municipality, 12°09′N 108°41′E, *L. Averyanov et al. VH2890, VH2914* (LE), 12°05′N 108°40′E, *L. Averyanov et al. VH2974* (HN, LE), 12°07′N 108°41′E, *L. Averyanov et al. VH3435* (LE, MO), 12°06′N 108°39′E, *L. Averyanov et al. VH4493* (HN, MO); **Lang Son Province**: *Sino-Vietnam Expedition 1597* (PE); **Lao Cai Province**: Sa Pa District, Hoang Lien National Park, *L.F. Fu et al. VMN_CN886* (IBK, VNMN); Van Ban District, 21°58′42″N 104°00′37″E, *L. Averyanov et al. HAL2094* (LE), 21°59′04″N 104°15′08″E, *L. Averyanov et al. HAL2094* (LE); **Nghe An Province**: Ky Son District, Na Ngoi Municipality, 19°11′52″N 104°10′55″E, *L. Averyanov et al. CPC6098* (LE); **Ninh Binh Province**: Nho Quan District, Phuoc Binh, 12°07′N 108°41′E, *L. Averyanov et al. VH3369* (HN, LE); **Quang Binh Province**: Minh Hoa District, Dan Hoa Municipality, 17°45′27″N 105°47′46″E, *L. Averyanov et al. HAL12539* (LE); **Quang Nam Province**: *L. Averyanov et al. VH919* (HN, LE); **Quang Ninh Province:** Ba Mun, 21°05′N 107°35′E, *L. Averyanov & E. Kudriavtseva 221* (LE); **Vinh Phuc Province**: *N. Arnautov et al. LX-VN3941* (LE); Tam Dao District, 21°28′02″N 105°38′39″E, *L.F. Fu et al. VMN_CN50* (IBK, VNMN); Tam Dao National Park, *N. Arnautov et al. LX-VN3859, LX-VN3920, LX-VN4045* (LE), *L. Averyanov 65-A* (LE).

**Elatostema ramosum** W.T.Wang

*Elatostema ramosum* var. *villosum* W.T.Wang.

China, Vietnam; 500–1,400 m.

**Cao Bang Province**: Nguyen Binh District, Yen Lac Municipality, 22°45′34″N 105°51′49″E, *L. Averyanov et al. CPC5424* (LE); **Ha Giang Province**: Bac Me District, Bac Me Nature Reserve, 22°45′14″N 105°13′36″E, *L.F. Fu et al. VMN_CN227* (IBK, VNMN); Quan Ba District, Bat Dai Son commune, Bat Dai Son Nature Reserve, San Chu village, 23°08′55″N 104°59′46″E, *L. Averyanov et al. VR065* (LE); **Hoa Binh** **Province**: Lac Son District, Tu Do Municipality, 20°25′29″N 105°19′36″E, *N.Q. Hieu et al. CPC1559* (LE); **Ninh Binh Province**: Cuc Phuong National Park, *T.H. Nguyen NTH 3015* (MO); Nho Quan District, Cuc Phuong National Park, *J.M. Hu Hu2065* (TAI).

**Elatostema retrohirtum** Dunn

China, Vietnam; 87–1,100 m.

**Bac Kan Province**: Na Ri District, Kim Hy Nature Reserve, 22°11′49″N 106°03′51″E, *L.F. Fu et al. VMN_CN120* (IBK, VNMN); **Gia Lai Province**: K’Bang District, Son Lang Municipality, Kon Chu Rang Nature Reserve, 14°30′55″N 108°32′48″E, *M.S. Nuraliev 2009* (MW); **Ha Giang Province**: Bac Me District, Bac Me Nature Reserve, 22°45′24″N 105°13′49″E, *L.F. Fu et al. VMN_CN217* (IBK, VNMN); between Meo Vac District and Dong Van District, *L.F. Fu et al. VMN_CN186*, *VMN_CN196*, *VMN_CN245* (IBK, VNMN); Quan Ba District, Tung Vai Municipality, *L.F. Fu et al. VMN_CN864* (IBK, VNMN); **Ha Noi City**: Ba Vi District, Ba Vi National Park, 21°04′22″N 105°21′36″E, *L.F. Fu et al. VMN_CN34* (IBK, VNMN), 21°03′35″N 105°21′50″E, *M.S. Nuraliev 1917* (IBK, MW); **Hai Phong City**: Cat Hai District, Cat Ba Island, Cat Ba National Park, Frog pond, 20°48.005′N 107°01.600′E, *N.A. Vislobokov 13030* (MW); **Hoa Binh Province**: Lac Son District, Ngoc Son Municipality, 20°26′31″N 105°19′32″E, *N.Q. Hieu et al. CPC1615* (LE); **Lam Dong Province**: Bao Loc, *L. Averyanov et al. LX-VN1713* (LE); **Nghe An Province:** Que Phong District, Hanh Dich Municipality, Pu Hoat Nature Reserve, 19°46′19″N 104°47′00″E, *M.S. Nuraliev 2180* (MW); **Ninh Binh Province**: Nho Quan District, Cuc Phuong National Park, *J.M. Hu Hu2099* (TAI); **Son La Province**: Phu Yen District, Gia Phu Municipality, Nhet Village, 21°36′27.7″N 104°04′22.8″E, *V.D. Nguyen et al. HNK2469* (K); **Tuyen Quang Province**: Na Hang District, Vinh Yen Municipality, 22°21′07″N 105°25′06″E, *N.T. Hiep et al. HLF-229* (HN).

**Elatostema rhizomatosum** (Gagnep.) Q.Lin

*Pellionia rhizomatosa* Gagnep.

Endemic; ca. 500 m.

**Province unknown**: Annam, col des Nuages prés Tourane, *M. Poilane 7909* (P).

**Elatostema rupestre** (Buch.-Ham. ex D.Don) Wedd.

*Procris rupestris* Buch.-Ham. ex D.Don.

*Elatostema zollingerianum* Miq.

India, Nepal, Vietnam; elevation unknown.

**Tuyen Quang Province**: Na Hang District, *A. Gramain et al. 649* (HN).

***Elatostema salvinioides** W.T.Wang

a. **Elatostema salvinioides** var. **robustum** W.T.Wang

China, Vietnam; 1,100–1,650 m.

**Dien Bien Province**: Muong Cha District, Mua Ngai Municipality, 21°53′46″N 103°10′07″E, *L. Averyanov et al. CPC1048, CPC1920* (LE); **Lai Chau Province**: Sin Ho District, Ta Ngau Municipality, 22°18′N 103°15′E, *N.T. Hiep et al. NTH2772* (LE).

**Elatostema scabrum** (Benth.) Hallier f.

*Pellionia scabra* Benth.

*Elatostema pellioniifolium* W.T.Wang, *Pellionia cephaloidea* W.T.Wang, *P. scabra* subvar. *pedunculata* Yamam., *Polychroa scabra* (Benth.) Hu*.*

China, Japan, Vietnam; 700–1,800 m.

**Hoa Binh Province**: Da Bac District, Cao Son Municipality, Buoi Truong station, 20°54′09″N 105°12′15″E, *N.T. Hiep et al. HAL592* (HN); **Kon Tum Province**: *L. Averyanov et al. VH255* (HN, LE, MO); **Phu Tho Province**: Tan Son District, Xuan Son Municipality, 21°06′49″N 104°56′03″E, *L. Averyanov et al. HAL12749* (LE); **Son La Province**: Yen Chau District, Muong Lum Municipality, 20°59′06″N 104°28′46″E, *D.K. Harder et al. 7298* (HN).

**Elatostema simplicissimum** Q.Lin

*Pellionia imbricata* Gagnep.

Endemic; 200–220 m.

**Lai Chau Province**: 22°03′N 103°09′E, *N.T. Hiep et al. NTH2747* (LE); Muong Lay District, Nam Hang Municipality, 22°08′14″N 102°58′18″E, *D.K. Harder et al. 5884* (K, LE).

**Elatostema sinense** H.Schroet.

*Elatostema sinense* var. *trilobatum* W.T.Wang*.*

China, Vietnam; ca. 500 m.

**Ha Giang Province**: Bac Me District, Bac Me Nature Reserve, *L.F. Fu et al. VMN_CN216A* (IBK, VNMN); Quan Ba District, Tung Vai Municipality, *L.F. Fu et al. VMN_CN872* (IBK, VNMN); **Hoa Binh Province**: Mai Chau District, Pa Co Municipality, *F. Wen et al. VMN_CN330, VMN_CN333* (IBK, VNMN); **Son La Province**: Bac Yen District, Ta Sua Municipality, Chu Village, 21°15′40.0″N 104°29′27.0″E, *V.D. Nguyen et al. HNK2724* (K).

**Elatostema sublineare** W.T.Wang

China, Vietnam; elevation unkown.

**Cao Bang Province**: Bao Lac District, Dinh Phung Municipality, *L.F. Fu et al. VMN_CN815* (IBK, VNMN); **Lang Son Province**: Van Lang District, *Anon. 1036* (LE); **Ninh Binh Province**: Nho Quan District, Cuc Phuong National Park, *J.M. Hu Hu2106* (TAI); **Tuyen Quang Province**: Na Hang District, *Anon. TV-74* (HN).

**Elatostema tenuicaudatum** W.T.Wang

China, Vietnam; 240–1,700 m.

**Cao Bang Province**: Bao Lac District, Xuan Truong Municipality, *L.F. Fu et al. VMN_CN808* (IBK, VNMN); **Dak Lak Province**: Krong Bong District, Cu Pui Municipality, 12°30′N 108°30′E, *L. Averyanov et al. VH6036* (HN); **Dien Bien Province**: Muong Cha District, Mua Ngai Municipality, 21°53′46″N 103°10′17″E, *L. Averyanov et al. CPC1921* (LE); **Gia Lai Province**: Mang Yang District, A Yun Municipality, Kon Ka Kinh National Park, 14°13′10″N 108°19′10″E, *M.S. Nuraliev et al. 1827* (IBK, MW); **Ha Giang Province**: Vi Xuyen District, Cao Bo Municipality, Tam Ve village, 22°46′03″N 104°49′42″E, *D.K. Harder et al. 5454* (MO), 22°46′12″N 104°49′33″E, *D.K. Harder et al. 5370* (MO); Yen Minh District, Du Gia Municipality, [23°56′N 103°13′E], *P.K. Loc et al. CBL2063* (LE); **Hoa Binh Province**: Lac Son District, Tu Do Municipality, 20°26′07″N 105°17′19″E, *N.Q. Hieu et al. CPC1499* (LE); **Khanh Hoa Province**: Khanh Son District, 12°12′N 108°44′E, *L. Averyanov et al. VH4313* (HN, LE, MO); **Lam Dong Province**: Lac Duong District, Da Chais Municipality, 12°09′N 108°41′E, *L. Averyanov et al. VH2891* (HN, LE, MO), 12°07′N 108°41′E, *L. Averyanov et al. VH3413* (LE), 12°11′N 108°43′E, *L. Averyanov et al. VH4387* (LE, MO); **Lao Cai Province**: Van Ban District, Khanh Yen Ha Municipality, 21°57′59″N 104°14′06″E, *L. Averyanov et al. HAL2462* (HN, LE); Van Ban District, Nam Xe Municipality, 22°01′27″N 104°00′25″E, *D.K. Harder et al. DKH7060* (LE); **Phu Tho Province**: Tan Son District, Xuan Son Municipality, 21°06′57″N 104°57′17″E, *L. Averyanov et al. HAL12639* (LE); **Quang Nam Province**: *L. Averyanov et al. VH905* (HN, LE); **Tuyen Quang Province**: Na Hang District, Vinh Yen Municipality, 22°21′09″N 105°25′20″E, *Le Van Cham et al. HLF-061* (HN); **Vinh Phuc Province**: Tam Dao District, 21°27′35″N 105°38′58″E, *L.F. Fu et al. VMN_CN45* (IBK, VNMN); Tam Dao Range, *N. Arnautov et al. LX-VN3921* (LE).

**Elatostema trichosanthum** (Gagnep.) H.Schroet.

*Pellionia trichosantha* Gagnep.

Endemic; elevation unknown.

**Ha Noi City**: Ba Vi District, Ba Vi Mountain, *B. Balansa 2529* (P).

**Elatostema tsoongii** (Merr.) H.Schroet.

*Polycharoa tsoongii* Merr.

*Pellionia cristulata* Gagnep.

Cambodia, China, Vietnam; 115–1,100 m.

a. **Elatostema tsoongii** ssp. **tsoongii**

**Bac Kan Province**: Na Ri District, Kim Hy Nature Reserve, 22°18′01″N 106°01′38″E, *L.F. Fu et al. VMN_CN139* (IBK, VNMN); **Dak Lak Province**: Krong Bong District, Cu Pui Municipality, 12°30′N 108°30′E, *L. Averyanov et al. VH5928* (HN); Lak District, Bong Krang Municipality, Chu Yang Sin National Park, 12°23′50″N 108°21′00″E, *M.S. Nuraliev 997* (K, MW); **Gia Lai Province**: Mang Yang District, A Yun Municipality, Kon Ka Kinh National Park, 14°12′53″N 108°18′56″E, *M.S. Nuraliev et al. 1449* (IBK, MW); **Ha Tinh Province**: Huong Son District, Son Hong Municipality, 18°34′06″N 105°11′40″E, *P.K. Loc et al. HAL5193* (HN, LE); **Lam Dong Province**: Bao Lam District, Lac Bac Municipality, 11°43′28″N 107°41′59″E, *M.S. Nuraliev et al. s.n.* (photo: MW); **Lang Son Province**: Huu Lung District, Huu Lien Municipality, Huu Lien Protected Area, 21°40′59″N 106°20′30″E, *D.K. Harder et al. 4120* (K); Pac Mo District, *Anon. 1149* (LE); **Quang Binh Province**: Minh Hoa District, Thong Hoa Municipality, vicinities of Yen Son Village, 17°40′N 105°57′E, *L. Averyanov et al. VH4729* (HN, LE, MO); **Quang Tri Province**: Da Krong District, Ta Rut Municipality, 16°24′29″N 107°01′07″E, *L. Averyanov et al. CPC3017* (LE); Lang Khoai, *Polilane 10741* (P); **Thanh Hoa Province**: Quan Hoa District, Pu Hu Nature Reserve, *F. Wen et al. VMN_CN352* (IBK, VNMN); **Thua Thien-Hue Province**: Phu Loc District, Bach Ma National Park, 16°14′11″N 107°44′28″E, *N.T. Hiep et al. HLF1649* (HN, MO); **Tuyen Quang Province**: Na Hang District, Thanh Tuong Municipality, near Ban Bung Village, 22°16′26″N 105°25′58″E, *P.K. Loc et al. HAL164* (HN); **Vinh Phuc Province**: Tam Dao National Park, *N. Arnautov et al. LX-VN4005* (LE).

b. **Elatostema tsoongii** ssp. **subpeltatum (**Gagnep.) H.W.Li

*Pellionia subpeltata* Gagnep.

China, Vietnam; elevation unknown.

**Bac Giang Province**: Lang Met, *P.A. Petelot 2929* (P).

**Elatostema veronicoides** (Gagnep.) H.Schroet.

*Pellionia veronicoides* Gagnep.

China, Vietnam; elevation unknown.

**Hoa Binh Province**: Da Bac District, Cho Bo, *B. Balansa 2251* (P); **Thanh Hoa Province**: Pu Huong Natural Reserve, *F. Wen et al. VMN_CN422* (IBK, VNMN).

**Elatostema vietnamense** Q.Lin & L.D.Duan

Endemic; elevation unknown.

**Thua Thien-Hue Province**: Phu Loc District, Bach Ma National Park, *H.N. Qin et al. 156* (PE).

**Elatostema xanthophyllum** W.T.Wang

China, Vietnam; 400–1,450 m.

**Bac Kan Province**: Na Ri District, Kim Hy Nature Reserve, 22°11′56″N 106°04′05″E, *L.F. Fu et al. VMN_CN134* (IBK, VNMN); Na Ri District, Kim Hy Nature Reserve, *Y.G. Wei & V.T. Do VMN_CN24* (IBK, VNMN); **Ha Giang Province**: Quan Ba District, 23°06′57″N 105°01′47″E, *D.K. Harder et al. 4830* (HN); Quan Ba District, Can Ti Municipality, 23°04′N 104°59′E, *N.T. Hiep et al. NTH3614* (K); Quan Ba District, San Chu village, 23°08′55″N 104°59′46″E, *L. Averyanov et al. VR063* (LE); Quan Ba District, Thanh Van commune, Mo Sai village, 23°07′46″N 104°57′56″E, *L. Averyanov et al. VR394* (LE); **Son La Province**: Van Ho District, Tan Xuan Municipality, 20°40′33″N 104°39′00″E, *L. Averyanov et al. CPC7117* (LE).

***Elatostema xichouense** W.T.Wang

China, Vietnam; 300–1,100 m.

**Ha Giang Province**: Quan Ba District, Bat Dai Son Nature Reserve, 23°09′N 104°59′E, *D.K. Harder et al. 5120* (HN), *L. Averyanov et al. VR052* (LE), *N.T. Hiep & L. Averyanov 5120* (MO); Quan Ba District, Bat Dai Son Nature Reserve, Thanh Van commune, Mo Sai village, 23°07′46″N 104°57′56″E, *L. Averyanov et al. VR365* (LE); Meo Vac District, *L.F. Fu et al. VMN_CN852* (IBK, VNMN); **Hoa Binh Province**: Lac Son District, Tu Do Municipality, 20°26′07″N 105°17′19″E, *N.Q. Hieu et al. CPC1482* (LE); Mai Chau District, Van Mai Municipality, 20°35′26″N 105°02′00″E, *D.K. Harder et al. 8170* (MO); **Ninh Binh Province**: Nho Quan District, Cuc Phuong National Park, *L. Averyanov et al. LX-VN1728* (LE).

**Excluded taxa**

**Elatostema cuneatum** Wight

*Elatostema approximatum* Wedd., *E. densiflorum* Franch. & Sav., *E. nipponicum* Makino, *E. webbianum* Wedd.

Bhutan, China, India, Indonesia, Laos, Thailand.

This species was indicated by Ho (2003) and Hiep (2005) as occurring in Vietnam but no specimen was cited and we were unable to locate any specimen from Vietnam.

## Additional Information and Declarations

### Data Availability

The following information was supplied regarding data availability: The raw data is available in the Appendix.
